# Stage II oesophageal carcinoma: peril in disguise associated with cellular reprogramming and oncogenesis regulated by pseudogenes

**DOI:** 10.1186/s12864-024-10023-9

**Published:** 2024-02-02

**Authors:** Govada Pravallika, Ramalingam Rajasekaran

**Affiliations:** Quantitative Biology Lab, Department of Integrative Biology, School of BioSciences and Technology, Vellore Institute of Technology, Vellore, Tamil Nadu India

**Keywords:** Differentiation, Pseudogenes, Cellular reprogramming, Oncogenic transformation, Oesophageal carcinoma, APOBEC mutations

## Abstract

**Introduction:**

Pseudogenes have been implicated for their role in regulating cellular differentiation and organismal development. However, their role in promoting cancer-associated differentiation has not been well-studied. This study explores the tumour landscape of oesophageal carcinoma to identify pseudogenes that may regulate events of differentiation to promote oncogenic transformation.

**Materials and method:**

De-regulated differentiation-associated pseudogenes were identified using DeSeq2 followed by ‘InteractiVenn’ analysis to identify their expression pattern. Gene expression dependent and independent enrichment analyses were performed with GSEA and ShinyGO, respectively, followed by quantification of cellular reprogramming, extent of differentiation and pleiotropy using three unique metrics. Stage-specific gene regulatory networks using Bayesian Network Splitting Average were generated, followed by network topology analysis. MEME, STREME and Tomtom were employed to identify transcription factors and miRNAs that play a regulatory role downstream of pseudogenes to initiate cellular reprogramming and further promote oncogenic transformation. The patient samples were stratified based on the expression pattern of pseudogenes, followed by GSEA, mutation analysis and survival analysis using GSEA, MAF and ‘survminer’, respectively.

**Results:**

Pseudogenes display a unique stage-wise expression pattern that characterizes stage II (SII) ESCA with a high rate of cellular reprogramming, degree of differentiation and pleiotropy. Gene regulatory network and associated topology indicate high robustness, thus validating high pleiotropy observed for SII. Pseudogene-regulated expression of *SOX2*, *FEV*, *PRRX1* and *TFAP2A* in SII may modulate cellular reprogramming and promote oncogenesis. Additionally, patient stratification-based mutational analysis in SII signifies *APOBEC3A* (A3A) as a potential hallmark of homeostatic mutational events of reprogrammed cells which in addition to de-regulated *APOBEC3G* leads to distinct events of hypermutations. Further enrichment analysis for both cohorts revealed the critical role of combinatorial expression of pseudogenes in cellular reprogramming. Finally, survival analysis reveals distinct genes that promote poor prognosis in SII ESCA and patient-stratified cohorts, thus providing valuable prognostic bio-markers along with markers of differentiation and oncogenesis for distinct landscapes of pseudogene expression.

**Conclusion:**

Pseudogenes associated with the events of differentiation potentially aid in the initiation of cellular reprogramming to facilitate oncogenic transformation, especially during SII ESCA. Despite a better overall survival of SII, patient stratification reveals combinatorial de-regulation of pseudogenes as a notable marker for a high degree of cellular differentiation with a unique mutational landscape.

**Supplementary Information:**

The online version contains supplementary material available at 10.1186/s12864-024-10023-9.

## Introduction

During organismal development, foregut endodermal specification leads to respiratory-oesophagus separation, wherein unique signalling events regulate epithelial differentiation and morphogenesis [1]. Albeit different, the signalling events associated with epithelial differentiation are essential during the adult human stage for the maintenance and repair of oesophagus that takes place approximately every 11 days. The considerably quick turnover of oesophageal epithelium warrants coordinated events of differentiation and proliferation of stem and progenitor cells of the basal layer. Aberrant tissue homeostasis has in fact, been associated with several oesophageal pathologies including eosinophilic oesophagitis (EoE) and Barrett’s oesophagus (BE) [2, 3].

BE is characterized by the transition of normal squamous epithelium to columnar epithelium as a result of chronic inflammation of the distal oesophagus [4]. Chronic inflammation, however, is in turn a manifestation of gastro-oesophageal reflux disease (GERD) that involves frequent reflux of gastric juices from the stomach to oesophagus. Gastric juices contain acid, bile salts and other noxious agents that induce tissue damage and repair in oesophagus during GERD, thus, promoting events of differentiation and proliferation [5]. BE-associated differentiation has been proposed to involve direct transdifferentiation, transdifferentiation via de-differentiation and progenitor cell reprogramming (transcommitment) [3, 4].

Metaplastic epithelium in BE is not only histologically distinct from adjacent tissue, but it also represents molecular changes that may promote precancerous events. In fact, BE harbours a mutational burden similar to that of several cancerous tissues and has a concurrent prevalence of approximately 91% with oesophageal adenocarcinoma (EAC), making it the only known precursor of EAC [6–9]. Furthermore, several differentiation-associated genes such as *c-Jun*, *cystatin A*, *SPRR3* and *PAX9* are dysregulated in oesophageal squamous cell carcinoma (ESCC); another sub-type of oesophageal cancer (ESCA) [10, 11]. Also, FGF2 has been implicated as a regulator of cancer stem-like cells (CSCs) in ESCC through Mek/Erk signalling [12]. While these studies have explored ESCA with differentiation as a critical component, the evidence is largely based on the study of coding genes.

Recent evidence, however, suggests that non-coding RNAs (ncRNAs) such as pseudogenes and long non-coding RNAs (lncRNAs) play a regulatory role in development and differentiation. *Olfr29-ps1,* for instance, has been proven to regulate differentiation and function of monocytic myeloid-derived suppressor cells to enhance their immunosuppressive potential within the tumour microenvironment [13]. Also, *POU5F1B*, an *OCT4* pseudogene is implicated in regulating the differentiation of human embryonic stem cells, further studied for its role in promoting HPV integration-driven cervical carcinogenesis and multidrug resistance (MDR) in chronic myeloid leukemia [14, 15]. While studies implicating the function of ncRNAs in regulating cancer-associated differentiation and several other maladies have gained momentum, their role in events of differentiation resembling the events of BE leading to oesophageal carcinoma is poorly understood.

Hence, we explored the tumour landscape of ESCA stage-wise to characterise the events of BE-type differentiation with respect to ncRNAs that may lead to distinct events of cellular reprogramming and oncogenic transformation in ESCA. Indeed, we report diverse stage-specific events of differentiation corresponding to the pattern of pseudogene and differentiation-associated coding gene expression. We further introduce two distinct metrics, cellular reprogramming and degree of differentiation to measure the extent of stage-specific differentiation. Also, we illustrate the potential of Stage II (SII) differentiation-associated genes in promoting oncogenic transformation through robustness of the gene regulatory network that concurs with a high degree of pleiotropy. Furthermore, the high degree of differentiation and cellular reprogramming observed within SII ESCA which is approximately equal to that of BE potentially implies that the precancerous state is more likely to transform into SII ESCA. We also report *SOX2*, a regulator of stem cell potential as a key transcription factor that potentially acts both downstream and upstream of DaPs to regulate cellular reprogramming and cancer progression in stage II ESCA.

Through a unique patient stratification followed by mutational analysis, we reveal the importance of APOBEC mutation-sequence preference in regulating the hypermutations in the background of combinatorial de-regulation of pseudogenes. We further define *APOBEC3A* as a potential hallmark of a homeostatic mutational landscape that better represents the reprogrammed cells in accordance with the high degree of differentiation observed for combinatorial expression of pseudogenes. While the overall survival of SII is better than that of its subsequent stages, unique degree of differentiation and mutational landscape for patient-stratified cohorts of SII makes it the most vulnerable stage of oesophageal carcinoma. Furthermore, we present pseudogenes involved in combinatorial expression and *APOBEC3A* as hallmarks of high degree of BE-type differentiation and cellular reprogramming, while *APOBEC3G* along with other survival-associated pseudogenes as hallmarks of oncogenesis and early-stage diagnosis.

## Materials and methods

### Data acquisition and availability

The open-source transcriptomic profiling (mRNA and miRNA), proteome profiling, clinical data and masked mutation annotation format (MAF) files are available on The Cancer Genome Atlas (TCGA) website (https://www.cancer.gov/tcga). The datasets for Oesophageal Carcinoma (ESCA, *n*=183) were filtered individually for patients with insufficient data on stage of cancer for downstream analysis, where each dataset contains *n*=151 patient samples except the miRNA dataset (*n*=149). The unstranded counts were used for downstream analysis as the transcriptomic data generated by TCGA for ESCA was not strand-specific.

### Screening for differentially expressed genes

The patient samples of transcriptomic profile data were stratified based on the pathological tumour stage of oesophageal carcinoma as described in the clinical data of TCGA-ESCA. For each mRNA and miRNA matrix, the analysis was performed in a group of two, namely; normal versus stage I (SI)/stage II (SII)/stage III (SIII)/stage IV (SIV). DESeq2 package (version 1.36.0) of R software was utilized to identify differentially expressed genes. A cut-off of adjusted *p*-value ≤ 0.05 and |log2FC| ≥ 1.5 was used as criteria to identify genes that significantly exhibit more than two-fold change in expression (either upregulation or downregulation). The expression intensity of the de-regulated genes was represented using the packages pheatmap (version 1.0.12) and ComplexHeatmap (version 2.12.0) based on Euclidean distance. The cluster analysis was performed using the default parameters of “ComplexHeatmap”.

### Screening for pseudogenes and coding genes associated with differentiation

Differentiation-associated genes were obtained from the GeneCards website (https://www.genecards.org) using "Differentiation" as the keyword. The relevant ‘Ensembl IDs’ were obtained from the Ensembl Database 108 (https://www.ensembl.org) using the BioMart tool. ‘Ensembl IDs’ of differentially expressed genes were compared with the IDs obtained from ‘Ensembl’ to identify differentiation-associated pseudogenes (DaPs) and differentiation-associated coding genes (DaCGs).

### Screening for pseudogene-interacting genes

The genes exhibiting known interactions with DaPs referred to as pseudogene-interacting genes (PiGs) in this article, were manually curated using NCBI Gene (https://www.ncbi.nlm.nih.gov/gene/) and PubMed (https://pubmed.ncbi.nlm.nih.gov). Both direct (assays performed in cell-lines or patient samples) and indirect interactions (bioinformatic analysis of experimental datasets) were used as eligible criteria for determining associated interactions. The expression intensity of PiGs was also represented using the packages pheatmap (version 1.0.12) and ComplexHeatmap (version 2.12.0) based on Euclidean distance. The clustering was performed using “ComplexHeatmap” with default parameters.

### Constitutive and stage-specific gene expression pattern

‘InteractiVenn’, a web-interface tool for Venn analysis was utilized to investigate the expression pattern of genes [16]. The term “Constitutive” is introduced in this study to represent the genes that are differentially expressed in all stages of ESCA. Thus,

$$Constitutive\ Gene\ Set={\bigcap }_{i=1}^{S}{GS}_{i}$$

$$Stage-specific\ Gene\ Set=\left\{\begin{array}{c}{GS}_{i}-{\sum }_{n=i+1}^{S}\left({GS}_{i}\cap {GS}_{n}\right) , if\ i=1\\ {GS}_{i}-\left(\left({\sum }_{n=i-1}^{1}\left({GS}_{i}\cap {GS}_{n}\right)\right)+\left({\sum }_{n=i+1}^{S}\left({GS}_{i}\cap {GS}_{n}\right)\right)\right) , if\ i>1\end{array}\right.$$

where,

*i* indicates pseudo-number assigned to stages of tumour landscape starting from the initial stage as 1

*S* indicates total number of stages

*GS* indicates Gene set of i^th^ or n^th^ stage

### Gene set enrichment analysis – expression dependent

Gene Set Enrichment Analysis was performed using GSEA software (version 4.2.1) for each stage of ESCA independently [17]. Briefly, the data matrix for analysis was sorted to include only normalized counts of DaPs, PiGs and DaCGs. Recommended parameters for ≥ 7 sample-size were used, which include 1000 permutations. Gene-set was used as the permutation type to identify gene sets that overlap that of non-oesophageal GI tract tissues.

Human Ensembl Gene ID was used as the Chip platform to match genes with their Ensembl ID (ftp.broadinstitute.org://pub/gsea/annotations_versioned/Human_Ensembl_Gene_ID_MSigDB.v2022.1.Hs.chip, accessed as on 3^rd^ Dec 2022). The gene ontology for biological processes (c5.go.bp.v2022.1.Hs.symbols.gmt), cell signature (c8.all.v2022.1.Hs.symbols.gmt), KEGG (c2.cp.kegg.v2022.1.Hs.symbols.gmt) and immune cell signatures (c7.all.v2022.1.Hs.symbols.gmt) were the modules used from GSEA-MSigDB to identify enriched pathways along with pathway-associated genes between normal and ESCA samples (stage-wise) [17, 18]. Top 30 pathways were identified with a gene-set size cut-off (max=100 and min=3). The enriched pathways were further filtered using a False Discovery Rate (FDR q-value) of ≤ 0.25, enrichment score (**|**ES**|**) of ≥ 0.50 and normalised enrichment score (**|**NES**|**) of ≥ 1.5.

### Leading-edge analysis

GSEA-derived Cell Signatures (CS-LEA), KEGG (KEGG-LEA), Gene Ontology-Biological Processes (GO_BP-LEA) and Immune Signatures (IS-LEA) were used for leading-edge analysis (LEA) using GSEA software as described below.

CS-LEA: Top 30-GSEA cellular signatures belonging to the post-foregut endodermal specification of embryo and adult stage were considered that represent non-oesophageal GI tract tissues, namely stomach, duodenum, small and large intestine. Cell signatures indicating the immune repertoire of non-oesophageal tissues were also considered.

KEGG-LEA: KEGG signatures with an FDR q-value ≤ 0.25 were identified only for stage II ESCA. These signatures were filtered for their association with cancer-related pathways.

GO_BP-LEA: Biological processes with an FDR q-value ≤ 0.25 associated with development, differentiation, polarity and cell-cell signalling were considered.

IS-LEA: Immune signatures with an FDR q-value ≤ 0.25 were identified only for stage III ESCA. All the signatures with significant FDR q-values were considered.

### Enriched degree of differentiation

The genes identified as a part of LEA for one enrichment module were analysed to identify the number of genes that exhibit overlap over more than one term or pathway within a module. We propose that genes enriched over multiple differentiation-associated terms within a module may indicate a higher likelihood of regulating differentiation. Hence, for a given stage the enriched degree of differentiation is defined as the average of the total number of overlapping genes divided by the total number of correlated terms or pathways for each enrichment module. Thus,

$$\text{Average Enriched Degree of Differentiation}=\frac{{\sum }_{i=1}^{N}\frac{\sum_{i=1}^{n}{G}_{i}}{P}}{N}$$

where,

*n* represents the total number of possible overlaps of terms or pathways

*G* represents number of genes exhibiting overlap for i^th^ overlap

*P* represents number of pathways that have overlapping genes

*N* is defined as the number of modules exhibiting enrichment for differentially expressed genes

### Gene set enrichment analysis – expression independent

The genes identified as a part of LEA were used to identify their role in promoting both differentiation as well as tumourigenesis due to their functional or genetic pleiotropy using ShinyGO [19]. The analysis was performed with default parameters for 6 different enrichment modules. For each module, the association between enriched pathways/terms (referred to as nodes) indicated by the overlap of genes was obtained. To identify “constitutive” as well as stage-specific pattern the enriched nodes were subject to ‘InteractiVenn’ analysis. The association between the filtered nodes was re-created using the R-package circlize (version 0.4.15). The network-related edges, nodes and their respective weights were obtained from ShinyGO analysis.

In addition to stage-specific and constitutive miRNAs identified from DESeq2 followed by ‘InteractiVenn’, multi-stage miRNAs were also identified using ShinyGO followed by comparison with differentially expressed miRNAs for appropriate cut-offs. The assumption for this approach is that miRNAs have diverse regulatory functions and thus, may reflect the same as multi-stage miRNAs.

### Degree of pleiotropy

The “constitutive” as well as stage-specific nodes were obtained from ‘InteractiVenn’ analysis and further investigated to identify the overlapping genes. For a given stage, the degree of pleiotropy is defined as the average of total number of overlapping genes divided by the total number of correlated terms/pathways for each enrichment module. Thus,

$$\text{Average Degree of Pleiotropy}= \frac{{\sum }_{i=1}^{M}\frac{\sum_{i=1}^{m}{G}_{i}}{F}}{M}$$

where,

*m* represents number of possible overlaps of two terms or pathways

*G* represents number of genes exhibiting overlap for i^th^ overlap

*P* represents number of pathways that have overlapping genes

*N* is defined as the number of datasets or modules exhibiting enrichment for differentially expressed genes

### Validation dataset

An independent gene expression dataset (GSE13898) was used to validate the aforementioned metrics including, degree of differentiation and cellular reprogramming [20]. The raw counts of microarray-based expression profile and clinical parameter files were downloaded from the GEO database (https://www.ncbi.nlm.nih.gov/geo/, accessed as on 29^th^ Nov 2023). The microarray expression profiles were normalized using the quantile normalization method as described previously using the Linear Models for Microarray Data (LIMMA) available in R [20, 21]. The normalized data was annotated for their respective phenotype labels and filtered further for analysis (Normal = 28, Barrett’s Oesophagus (BE) = 15, Stage I = 11 with 3 duplicate array profiles, Stage II = 23 with 3 duplicate array profiles, Stage III = 7 with 1 duplicate array profile). For GSEA, duplicate array profiles were considered as independent samples with the same clinical characteristics as previously described by Kim et al. during heat-map clustering [20].

The Gene Set Enrichment Analysis was performed using GSEA software (version 4.3.2) for each stage independently against normal samples [17]. The entire microarray-based expression matrix was used for GSEA analysis to identify if cell signatures of non-oesophageal GI tract tissues are significantly enriched without a priori knowledge of gene association with the term “differentiation”. Hence, default parameters of GSEA were used, which include 1000 permutations with phenotype as permutation type with a gene-set size cut-off (max=500 and min=15). Human Illumina Array was used as Chip platform to match genes with their respective probe-IDs (ftp.broadinstitute.org://pub/gsea/msigdb/human/annotations/Human_ILLUMINA_Array_MSigDB.v2023.2.Hs.chip, accessed as on 9^th^ Dec 2023). Cell signature (c8.all.v2023.2.Hs.symbols.gmt) was used to identify enriched signatures along with signature-associated genes between normal and ESCA (stage-wise) or BE samples.

The enriched signatures were filtered using an FDR q-value of ≤ 0.25 and |NES| ≥ 1.3 (minimum absolute value of non-oesophageal GI tract signatures observed in Normal vs BE enrichment). LEA using GSEA was performed as described before in the previous sections for all non-oesophageal GI tract Cell-Signatures.

### Gene regulatory network construction and analysis

Bayesian network analysis with splitting average strategy (BNSA) was employed to generate stage-specific gene regulatory networks (GRN) [22]. Briefly, the stage-specific data matrix used for GSEA was discretized using binary values for an individual gene as described previously with 0 for below-median expression and 1 for above-median expression. The ‘Hillclimbsearch’ method with ‘BIC’ scoring was used to generate networks and their respective adjacency matrices. The adjacency matrix obtained from BNSA consisting of nodes and edges was used to recreate GRNs using Cytoscape (version 3.9.1). Briefly, z-scores generated from normalized counts of cancer samples were used to generate scatter plots of co-expression using the ggpubr (version 0.5.0) and ggplot2 (version 3.4.0) packages. The co-expression plots were additionally filtered using Pearson and Spearman correlation scores using a *p*-value cut-off of 0.05.

The edges of GRNs were re-created with edges of varying thickness based on the *p*-value cut-off. In brief, edges with lower thickness represent a significant *p*-value only for either Spearman or Pearson correlation coefficient, medium thickness represents a significant *p*-value for both coefficients with < 0.89 for absolute values of correlation and highest thickness represents a significant *p*-value for both coefficients with ≥ 0.89 for absolute values of correlation coefficient.

The ‘Original Network’ (ON) represents all the genes as nodes with their respective connections identified from BNSA. The ‘Degree of differentiation’ (DD) network was recreated using ON to represent all DaPs and miRNAs of ON along with overlapping genes that are identified only as a part of GSEA-LEA. The ‘Degree of pleiotropy’ (DP) network was recreated using ON to represent all DaPs and miRNAs along with overlapping genes identified from Expression-Independent Enrichment analysis.

Network analysis of GRNs was performed using Cytoscape (version 3.9.1) to identify the characteristics of the GRNs. In brief, before network analysis self-loops were removed while multiple edges were retained if the type of interactions were different. The network parameters graph density, betweenness centrality, closeness centrality, eigenvector centrality, degree centrality and subgraph centrality were computed by retaining or removing pseudogenes for ON, DD and DP-networks individually.

### *De novo* transcription factor and miRNA motif analysis (stage II)

Transcription factor (TF) and miRNA motif analysis were performed for SII ESCA genes using MEME Suite (version 5.5.2; accessed as on 11^th^ April 2023) using the tools ‘Simple, Thorough, Rapid, Enriched Motif Elicitation’ (STREME) and ‘Multiple Em for Motif Elicitation’ (MEME) [23, 24]. Both relative enrichment analysis and independent enrichment analysis were performed. For relative enrichment of TF and miRNA motifs in DaPs with respect to non-TF coding genes using STREME, upregulated and downregulated gene sequences were used as control sequences in two separate analyses. For both MEME and STREME, the 1^st^ Order Markov Model of sequences were used as the background with default *E-value* (<10) and *p-value* (0.05) parameters. The minimum width of motifs was set to 8 while the maximum width was set to 30. The sequences for analysis were pre-processed as described below.

In brief, non-TF coding genes having putative or known associations with TFs identified as a part of GRN were filtered and their respective sequences were obtained from NCBI Gene (accessed as on 3^rd^ April 2023). The truncated sequences suitable for STREME analysis were generated using transcription start site (TSS) information obtained from Ensembl Database 109 (accessed as on 7^th^ April 2023). The truncated sequences consist of TSS positioned in the centre with a 2000 base pair (bp) region extending on either side of the TSS. However, sequences with TSS positioned either in the gene start or end were modified to include 4000 bp extensions towards a single side of TSS (forward and backward for TSS at gene start and end, respectively) to maintain a uniform set of primary sequences supplied to STREME.

For pseudogenes, the truncated sequences for STREME were generated based on the length of the smallest sequence (herein, *IGHV3-71* with 462bp). For a given gene, each consecutive sequence had an overlap of 52 base pair region with the previous sequence to ensure that putative motifs near truncated regions were also identified.

For TF genes, the sequences were truncated as described for non-TF coding genes. MEME instead of STREME was used for analysis as the total number of sequences was less than 50. Zero or One occurrence per sequence was used with the rest of the parameters as described before.

All *de novo* motifs with E-value ≤ 1.0 and *p*-value ≤ 0.05 were further subject to known motif analysis using ‘Tomtom’ across the databases Eukaryote DNA (Vertebrates (*In vivo* and *in silico*)), HUMAN (*Homo sapiens*) DNA (HOCOMOCO Human v11 CORE) and miRBase (v22) Single Species microRNA (DNA-encoded) for Homo_sapiens_hsa (DNA-encoded). For all Tomtom analyses, default parameters were used. The results were filtered according to FDR q-value ≤ 0.25 (25%) and *p*-value ≤ 0.05. The filtered motifs were then compared with the presence of differentially expressed genes that are a part of SII GRN.

Motif Alignment and Search Tool (MAST (version 5.5.2; accessed as on 15^th^ April 2023)) was further used with default parameters to identify motif sites, their alignment as normal as well as reverse complement and their respective *p-values* [25].

### Mutation landscape analysis of ESCA-TL

MAF Tools was used to analyse the mutation landscape of ESCA from MAF files as described before [26]. To generate rainfall plots representing kataegis for varied gene expression patterns of the APOBEC superfamily across stage II ESCA, the data matrix was segregated based on the median expression values. Patients with higher than median expression were assigned as positive, while patients with lower than median expression values were assigned as negative. The MAF data matrices thus generated were used for individual kataegis analysis. The average inter-mutation distances were obtained from the same.

### Mutational landscape and degree of differentiation for combinatorial expression of DaPs in SII ESCA

The data matrices for mutation analysis across combinatorial gene expression of DaPs belonging to the SII gene regulatory network (represented as UDaP_Combo for Upregulated & downregulated DaPs Combination and vice versa) were generated by segregating them based on gene expression values of DaPs. Both constitutive as well as stage-specific genes were considered for data matrix segregation. In brief, the gene expression values of DaPs were binarized as described for GRN. These values were further used to calculate the median of upregulation for upregulated DaPs and likewise for downregulated DaPs across each patient sample. The patient samples with a median value of all DaP-upregulation (median-DaP_up_) ≥ 0.5 indicate most genes with Log2FC ≥ 1.5 are in their upregulated state while a median value of all DaP-downregulation (median-DaP_down_) = 0 indicates that most genes with Log2FC ≤ -1.5 are in their downregulated state for a given sample. Thus, for a given patient sample the combination of median-DaP_up_ ≥ 0.5 and median-DaP_down_ = 0 is discretized as Yes for UDaP_Combo and vice versa. These data matrices were further used for mutational landscape analysis as mentioned before.

The ‘Degree of Differentiation’ for the two cohorts was calculated as described before using GSEA. Briefly, two different data matrices for the aforementioned cohorts (normal vs UDaP_Combo and normal vs UDaP_No_Combo) were generated to include normalized counts of all differentially expressed genes. Recommended parameters for > 7 sample size were used, which include 1000 permutations for phenotype as permutation type to identify gene-sets that significantly overlap that of non-oesophageal GI tract tissues. Human Ensembl Gene ID was used as Chip platform to match genes with their Ensembl ID (ftp.broadinstitute.org://pub/gsea/annotations_versioned/Human_Ensembl_Gene_ID_MSigDB.v2022.1.Hs.chip, accessed as on 10^th^ May 2023). Cell signature (c8.all.v2022.1.Hs.symbols.gmt) was used to identify enriched cell signatures along with signature-associated genes between normal and stratified cohorts (either UDaP_Combo or UDaP_No_Combo). Top 50 pathways were identified with a gene-set size cut-off of max=100 and min=3. The enriched pathways were further filtered using a False Discovery Rate (FDR q-value) of ≤ 0.25, enrichment score (|ES|) of ≥ 0.40 and normalised enrichment score (|NES|) of ≥ 1.5.

### CoxPH regression and survival analysis

The right-censored data for survival (in days) was obtained from the TCGA database using the ‘RTCGA.clinical’ package (version 20151101.26.0) and the data was further left-censored for unknown time of diagnosis or negative time of diagnosis. Cox Proportional Hazards regression and survival analysis were performed using the package ‘survival’ (version 3.5-3) and ‘survminer’ (version 0.4.9). The median of normalized expression values was used as the cut-off for discretizing the expression values as ‘High’ for above median and ‘Low’ for below median. *p-value* ≤ 0.05 was used as the cut-off for coxph likelihood test and survival analysis.

### Statistical analysis and plots

All relevant statistical analysis was performed using GraphPad Prism Licensed Software (version 9.5.1) unless obtained as a part of a specific R/python/web tool used for aforementioned analyses. The expression-associated box and whisker plots and volcano plots were generated using GraphPad. *p*-value ≤ 0.05 was used as a cut-off for all analyses, unless otherwise mentioned.

## Results

### Differentiation-associated pseudogenes have diverse expression pattern across oesophageal carcinoma

**Fig. 1:**
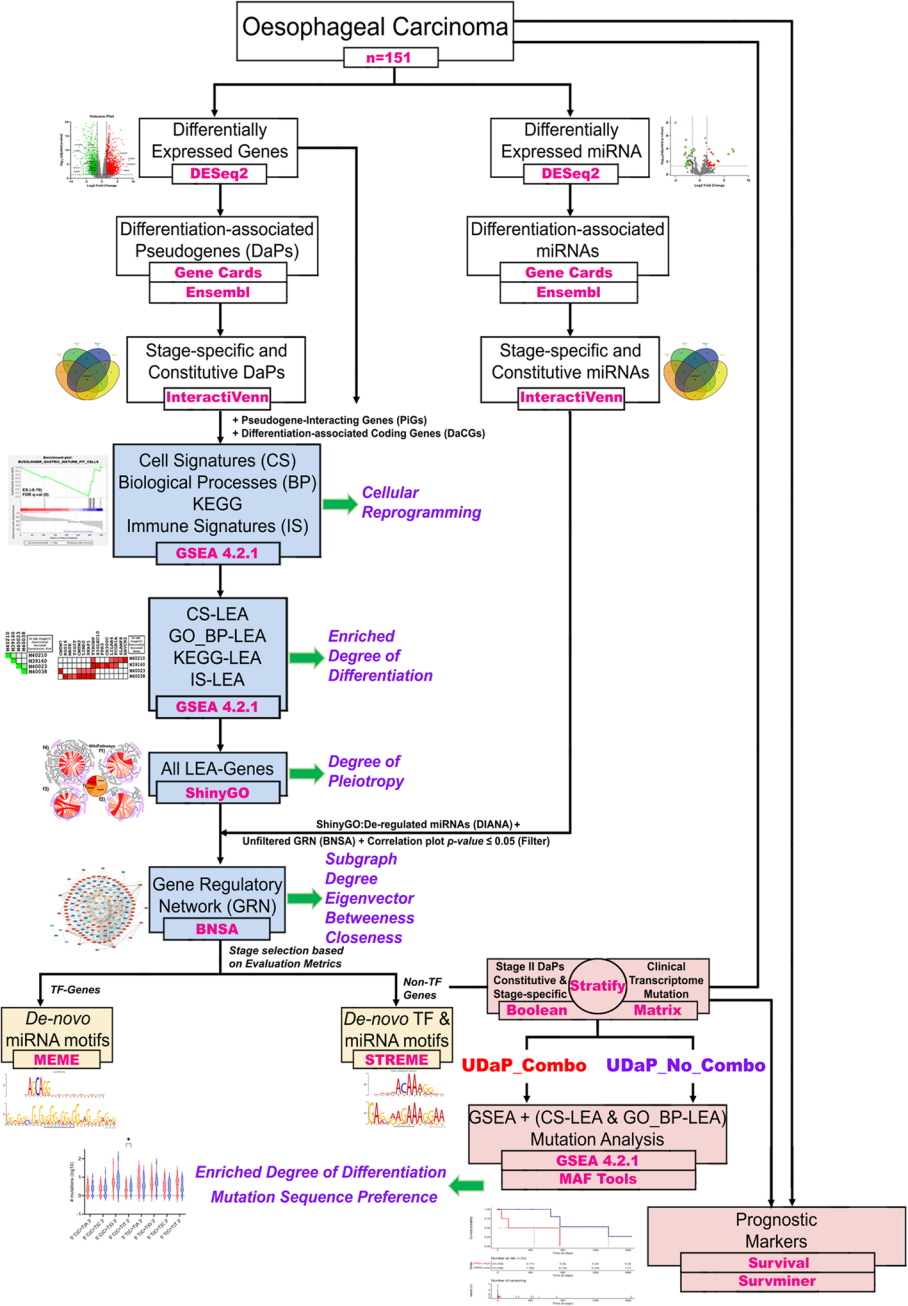
Flowchart indicating the workflow to identify unconventional biomarkers of ESCA The tumor landscape of ESCA is subject to the modules as depicted. The modules in white indicate the primary steps of our analysis to identify stage-specific and constitutive genes associated with differentiation. The modules in blue indicate the analyses where unconventional metrics were designed and used to identify the extent of cell reprogramming, differentiation and pleiotropy. Each metric associated with a given module is highlighted in purple with a green arrow. Post gene regulatory network analysis, the DaPs of stage II were utilised to uniquely stratify the stage II samples using a Boolean matrix. These stratified samples are subject to individual DESeq2 analysis followed by GSEA as well as mutational analysis. The cohort highlighted in red indicates an unorthodox hallmark of high differentiation and cellular reprogramming and unique mutational landscape. The stratified samples along with all other stages of ESCA are subject to survival analysis. For each module, the tools used for analysis are highlighted in pink (bold), while the primary results obtained as a part of the module are in black

**Fig. 2:**
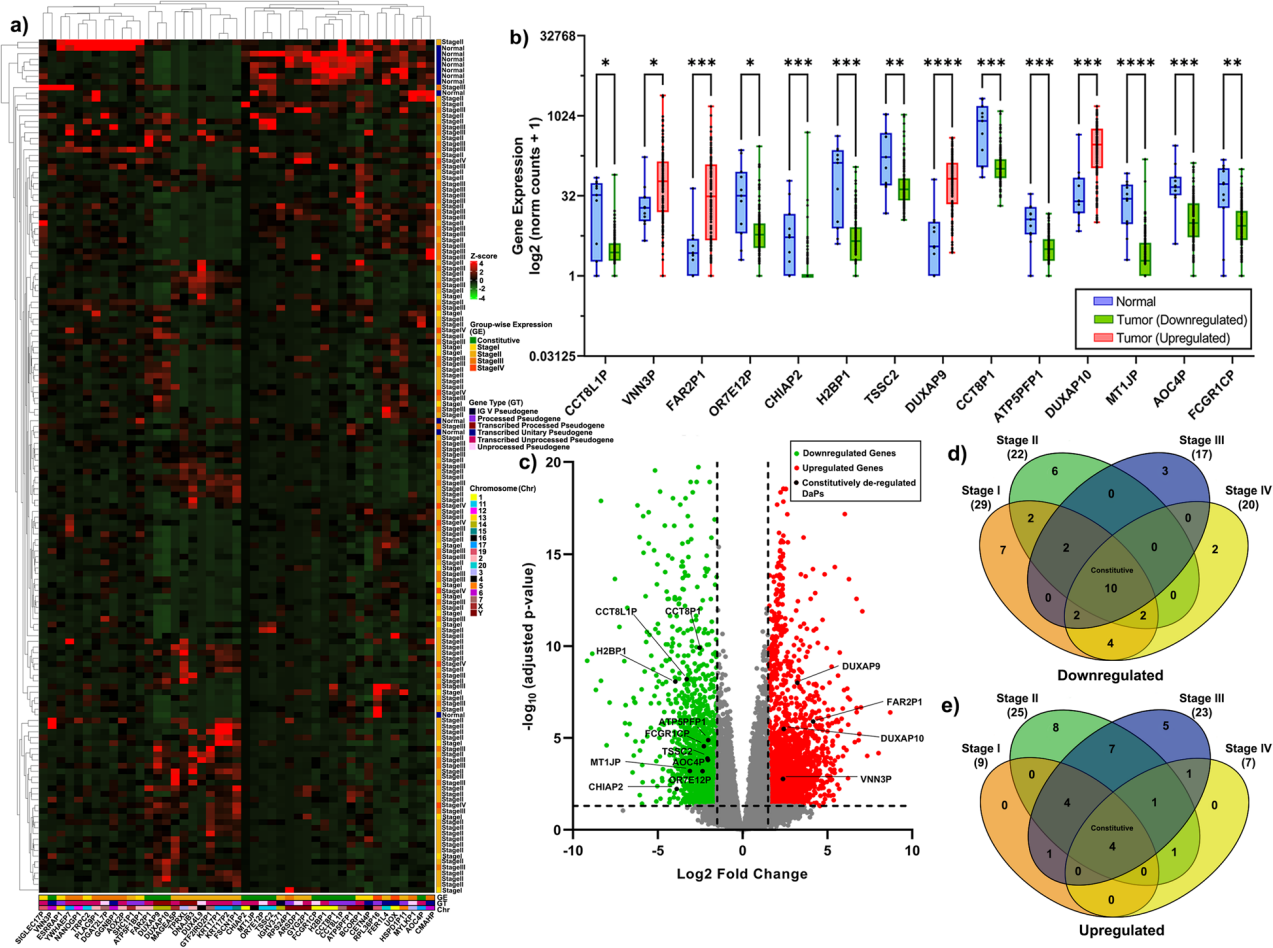
Differentiation-associated pseudogenes (DaPs) have Diverse Expression Pattern across ESCA. **a** Clustered heatmap of DaPs representing the differential expression pattern observed in ESCA, normalized across all samples by z-score; green indicates downregulation while red indicates upregulation. The right annotation represents clustered data by group (indicated by the cluster dendrogram on left) identified by blue, yellow, gold, orange and orange-red for patient samples stratified as normal, Stage I, Stage II, Stage III and Stage IV, respectively. The bottom annotation represents clustered DaPs by group-wise expression (indicated by cluster dendrogram on top). Additionally, the bottom annotation indicates the type of DaP and chromosomal location **b** Box and whisker plot indicating de-regulated DaPs between normal and cancer groups that exhibit constitutive expression pattern. Significance of expression between the two groups was evaluated using Mann-Whitney tests, indicated using asterisks above the boxplot as ****p < 0.0001, ***p < 0.001, **p < 0.01 and *p < 0.05. **c** Volcano plot of differentially expressed DaPs indicating down-regulation as green and up-regulation as red dots. The dashed lines along x-axis and y-axis represent cut-off of |log2FC| ≥ 1.5 and cut-off of -log10(adjusted *p*-value) > 1.30103, respectively. Genes with adjusted *p*-value < -log10(0.05) and/or |log2FC| < 1.5 are indicated by grey dots. **d**-**e** Venn diagrams of downregulated (**d**) and upregulated (**e**) DaPs in each stage compared to that of normal individuals. The overlapping portion indicated by the term “Constitutive” represents DaPs de-regulated in all stages of ESCA with a uniform expression pattern. DaP; Differentiation-associated pseudogenes, FC; Fold Change and ESCA; Oesophageal Carcinoma

The workflow as described in Fig. 1 was implemented to identify differentiation-associated pseudogenes (DaPs) that potentially regulate the events of differentiation and induce oncogenesis across various stages of Oesophageal Carcinoma (ESCA). Differentially expressed genes were identified using DESeq2 and additionally filtered for DaPs using ‘Gene Cards’ based on the search term “Differentiation”. Further, expression pattern analysis using ‘InteractiVenn’ indicated 45 DaPs with distinct expression profiles (Fig. 2a, d, e and Table 1). Among the 45 DaPs, 14 are ‘Constitutively’ de-regulated of which, 4 are upregulated (Fig. 2b-c, e) while 10 are downregulated (Fig. 2b-d). The remaining 31 DaPs exhibit stage-specific expression profile (Fig. 2a, d-e), among which 13 exhibit upregulation while 18 exhibit downregulation. Of the stage-specifically upregulated DaPs, 8 belong to Stage II (SII) (Fig. S1c-d) and 5 to Stage III (SIII) (Fig. S1e-f). Similarly, of the stage-specifically downregulated DaPs, 7, 6, 3 and 2 belong to stages I, II, III and IV, respectively.

**Table 1:** Differentially expressed differentiation-associated pseudogenes (DaPs) in Oesophageal Carcinoma

**Gene**	**Expression Profile**	**ENSEMBL Id**	**Stage I**	**Stage II**	**Stage III**	**Stage IV**
*AOC4P*	Downregulated	ENSG00000260105	YES	YES	YES	YES
*AOX2P*	Upregulated	ENSG00000243478	-	-	YES	-
*ARSDP1*	Downregulated	ENSG00000225117	-	YES	-	-
*ATP5F1BP1*	Downregulated	ENSG00000231635	YES	-	-	-
*ATP5PFP1*	Downregulated	ENSG00000237701	YES	YES	YES	YES
*BCORP1*	Downregulated	ENSG00000215580	YES	-	-	-
*CCT8L1P*	Downregulated	ENSG00000020219	YES	YES	YES	YES
*CCT8P1*	Downregulated	ENSG00000226015	YES	YES	YES	YES
*CETN4P*	Downregulated	ENSG00000224786	YES	-	-	-
*CHIAP2*	Downregulated	ENSG00000203878	YES	YES	YES	YES
*CMAHP*	Downregulated	ENSG00000168405	YES	-	-	-
*DGAT2L7P*	Upregulated	ENSG00000205267	-	-	YES	-
*DNAJB3*	Upregulated	ENSG00000227802	-	YES	-	-
*DUX4L9*	Upregulated	ENSG00000224807	-	YES	-	-
*DUXAP10*	Upregulated	ENSG00000244306	YES	YES	YES	YES
*DUXAP9*	Upregulated	ENSG00000225210	YES	YES	YES	YES
*ESRRAP1*	Downregulated	ENSG00000215572	YES	-	-	-
*FAR2P1*	Upregulated	ENSG00000180178	YES	YES	YES	YES
*FCGR1CP*	Downregulated	ENSG00000265531	YES	YES	YES	YES
*FER1L4*	Downregulated	ENSG00000088340	-	YES	-	-
*FSCN1P1*	Upregulated	ENSG00000261559	-	YES	-	-
*GGNBP1*	Upregulated	ENSG00000204188	-	-	YES	-
*GTF2IRD2P1*	Upregulated	ENSG00000214544	-	YES	-	-
*GYG2P1*	Downregulated	ENSG00000206159	-	YES	-	-
*H2BP1*	Downregulated	ENSG00000223345	YES	YES	YES	YES
*HSPD1P11*	Downregulated	ENSG00000251348	-	YES	-	-
*IGHV3-71*	Downregulated	ENSG00000254056	-	YES	-	-
*KRT17P1*	Upregulated	ENSG00000131885	-	YES	-	-
*KRT17P2*	Upregulated	ENSG00000186831	-	YES	-	-
*MAGEA5P*	Upregulated	ENSG00000242520	-	YES	-	-
*MT1JP*	Downregulated	ENSG00000255986	YES	YES	YES	YES
*MYLKP1*	Downregulated	ENSG00000228868	-	-	YES	-
*NANOGP1*	Upregulated	ENSG00000176654	-	-	YES	-
*OR7E12P*	Downregulated	ENSG00000189398	YES	YES	YES	YES
*PLAC9P1*	Downregulated	ENSG00000214100	-	-	YES	-
*RPL36P16*	Downregulated	ENSG00000224476	-	-	-	YES
*RPS24P1*	Downregulated	ENSG00000236477	-	-	YES	-
*SHC1P1*	Downregulated	ENSG00000230889	-	YES	-	-
*SIGLEC17P*	Downregulated	ENSG00000171101	YES	-	-	-
*TPRXL*	Upregulated	ENSG00000180438	-	YES	-	-
*TRPC2*	Downregulated	ENSG00000182048	YES	-	-	-
*TSSC2*	Downregulated	ENSG00000223756	YES	YES	YES	YES
*UOX*	Downregulated	ENSG00000240520	-	-	-	YES
*VNN3P*	Upregulated	ENSG00000093134	YES	YES	YES	YES
*YWHAEP7*	Upregulated	ENSG00000276715	-	-	YES	-

To further understand the role of DaPs in regulating the events of differentiation, we curated known interactors of DaPs using ‘PubMed’ and ‘NCBI Gene’ (Tables S1 and S2). Interestingly, only 13.3% of the curated genes (36 out of 270) were identified to be differentially expressed with 25 being upregulated, while 11 were downregulated (Table S3). Although, DaP-interacting genes (PiGs) exhibit a diverse expression pattern similar to that of DaPs, only 8 (22.2%) of the 36 PiGs complement and coincide with the expression pattern of their respective interacting DaPs. Among the complementary PiG-DaP interactions, 7 PiG-DaPs exhibit constitutive expression pattern (Fig. S2a, d, g and j), while only *PAX7-DUX4L9* axes exhibit SII-specific expression (Fig. S2d).

Several non-coding genes, including pseudogenes and miRNAs, demonstrate tissue-specific expression with diverse interacting partners [27]. Thus, while we curated and analysed PiGs in ESCA to find the presence of differential expression of known interacting partners, we also analysed other differentiation-associated coding genes (DaCGs) to identify potential novel binding partners. Interestingly, the differential expression pattern of DaCGs was highly stage-specific (Fig. S2b, e, h and k) with no overlap across any of the four stages of ESCA. Our analysis, thus, indicates that DaPs have a unique expression pattern in ESCA. Albeit limited in number, DaP-PiG interactions may regulate all stages of ESCA uniformly, while DaCGs may regulate tumourigenesis by acting independently or in association with DaP-PiG axes to drive distinct stage-specific events.

### Gene set enrichment analysis characterizes ESCA with heterogenous non-oesophageal cell signatures of adult and embryonic stages

**Fig. 3:**
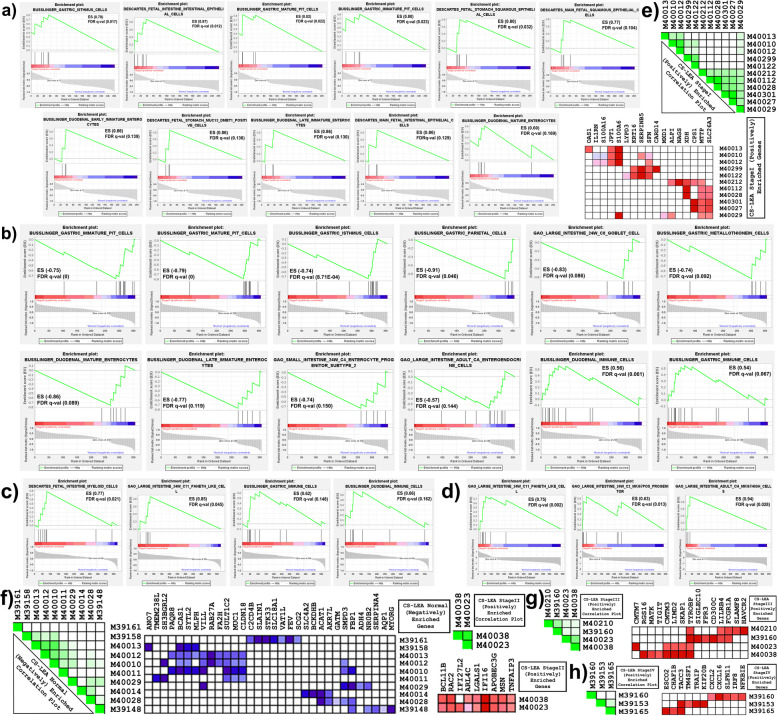
Cellular Signatures representing adjacent tissues of oesophagus are enriched for differentially expressed PiGs and DaCGs **a**-**d** GSEA plots sorted by highest to lowest normalized enrichment score (NES) indicating the enrichment for de-regulated PiGs and DaCGs for Stage I (**a**), Stage II (**b**), Stage III (**c**) and Stage IV (**d**) ESCA. For each plot, the enriched dataset is indicated above the plot as a label. The x-axis represents the Enrichment Score (ES), while the y-axis represents the correlation of differential expression of genes for samples as a gradient bar with red for highest correlation and blue for lowest. Each gene is represented by a vertical line labelled as ‘hits’ along the y-axis with a representation of the associated signal-to-noise ratio vs rank in the dataset. ES and FDR q-val are indicated for each plot in either the top right corner or bottom left corner. **e**-**h** LEA plots indicating the correlation between the enriched cellular signatures with their respective GSEA IDs and heatmaps indicating the differential expression of genes enriched for Stage I (**e**), Stage II (**f**), Stage III (**g**) and Stage IV (**h**) ESCA. For heatmap, the GSEA IDs are indicated on the right-side, while differentially expressed genes are indicated above the heatmap. GSEA; Gene Set Enrichment Analysis, PiGs; DaP-interacting genes, DaPs; Differentiation-associated pseudogenes, DaCGs; Differentiation-associated coding genes, LEA; Leading Edge Analysis, CS-LEA; Cell Signatures-Leading Edge Analysis, FDR q-val; False Discovery Rate q-value and ESCA; Oesophageal Carcinoma

Aberrant events of transdifferentiation and transcommitment during oesophageal tissue homeostasis can lead to metaplastic epithelium with diverse cell signatures (CSigns) representing adjacent tissues of the GI tract [2–4]. Thus, we employed gene-set enrichment analysis using GSEA software for DaPs, PiGs and DaCGs to identify the enrichment of such CSigns in ESCA that represent non-oesophageal GI tract (referred to as GI^Non-Oesophageal^) tissues. CSign analysis indicated that for Stage IV (SIV) and SIII ESCA, the GI^Non-Oesophageal^ tissues represented only a small fraction of the top30 CSigns despite being advanced stages of ESCA (10% and 6.66%, respectively) (Fig. 3c-d, Table S4). However, stage I (SI) and SII had comparatively higher fractions of the top 30 CSigns that represented GI^Non-Oesophageal^ tissues (37.93% and 22.67%, respectively) (Fig. 3a-b and Table S4). It was interesting to note that across all stages of ESCA, several CSigns represented lung cells and lung-associated immune cells (Table S4) that are characterised as adult respiratory tract cells [28]. To identify if these results can be recapitulated on an independent dataset, GSEA for CSigns was performed on ‘GSE13898’ which included samples of BE as well [20]. As expected, GI^Non-Oesophageal^ as well as lung CSigns were observed not only across all stages of EAC but also samples of BE thus, indicating that transdifferentiation and transcommitment indeed play a significant role during BE with the formation of metaplastic epithelium that finally promotes tumourigenesis (Table S4).

Among the GI^Non-Oesophageal^ signatures, stomach and duodenum signatures (SD-CSigns) have been characterized as adult cells while signatures of small and large intestine (SL-CSigns) are characterized as embryonic cells [29–31]. Given the diversity of CSigns within each stage, we propose that these signatures indirectly represent the rate of cellular reprogramming. Thus, we examined the ratio of SD-CSigns to SL-CSigns (SD/SL), where a higher ratio corresponds to faster transformation to non-oesophageal adult cells and vice versa. Indeed, we identified distinct SD/SL ratios where, it was lowest for SIV (0), followed by SIII (1) and SI (1.2) and highest for SII (3), thus indicating a faster rate of cellular reprogramming for SII as compared to other stages. Furthermore, an identical analysis on ‘GSE13898’ implicated that the extent of cellular reprogramming was in fact, highest for BE followed by SII EAC which was marginally higher than that of SIII EAC (Table S4).

In addition to cell signatures analysis, GSEA was further utilised to identify biological processes that potentially indicate events of development, differentiation and epithelial signalling. As expected, for all stages we identified biological processes associated with differentiation (Table S4, Fig. S3a-b, d, f). It was interesting to note that, only for SII, several processes associated with a change in the polarization and/or projection of cells were enriched indicating structural re-organization of cells (Table S4). Structural re-organization has been associated with the differentiation of stem cells as well as epithelial integrity, thus, suggesting that the enriched biological processes may in fact, reinforce the high rate of cellular reprogramming observed in SII [32–34].

### Unique stage II tumour landscape is associated with high degree of differentiation and pleiotropy

The distinct rate of cellular reprogramming prompted us to characterise the extent of differentiation by analysing the genes that regulate multiple GSEA terms, where a higher overlap of genes indicates a higher likelihood of their function as differentiation-associated genes. We performed ‘Leading Edge Analysis’ (LEA) for each module to identify such overlapping genes (Fig. 3e-h and Fig. S3). For a given module, the LEA-identified genes were divided by the number of correlated terms to obtain ‘Enriched Degree of Differentiation’. We observed that the average enriched degree of differentiation was highest for SII which concurs with the high rate of cellular reprogramming (Table 2). Furthermore, a similar analysis for GI^Non-Oesophageal^ signatures on ‘GSE13898’ revealed that the enriched degree of differentiation is not only the highest for SII but also approximately equal to that of BE, followed by SIII and SI (Table S4).

**Table 2:** Table indicating average degree of differentiation for each stage. In brief, enriched degree of differentiation is obtained by dividing the number of genes that exhibit overlap over more than one term or pathway within a module divided by the number of nodes indicating correlation within a given module separately and then averaged over all modules for a given ESCA-stage. The terms, processes and pathways are referred to as nodes. ESCA; Oesophageal Carcinoma, NES; Normalised Enrichment Score, LEA; Leading Edge Analysis, CS-LEA; Cell Signatures-LEA, GO_BP; Gene Ontology_Biological Processes-LEA, IS-LEA; Immune Signatures-LEA, KEGG-LEA; Kyoto Encyclopaedia of Genes and Genomes-LEA and GSEA; Gene Set Enrichment Analysis

**ESCA Stage**	**Enriched Degree of Differentiation**				
	**CS-LEA Genes (Terms)**	**GO_BP-LEA Genes (Processes)**	**IS-LEA Genes (Terms)**	**KEGG-LEA Genes (Pathways)**	**Average**
**Stage I**	12 (11)	7 (4)	-	-	1.420
**Stage II**	24 (10)	37 (9)	-	8 (5)	2.704
**Stage III**	6 (4)	-	17 (16)	-	1.281
**Stage IV**	1 (3)	10 (4)	-	-	1.416

Genetic and functional pleiotropy aids in the regulation of multiple processes and signalling events across several organs [35]. Hence, we explored the role of PiGs and DaCGs (from GSEA-LEA) in regulating non-differentiation-associated pathways by performing expression-independent GSEA using ShinyGO over six different modules. Indeed, we identified the enrichment of non-differentiation-associated processes (referred to as nodes) across all modules signifying their functional pleiotropy (Fig. S4a1-f4). Furthermore, ‘InteractiVenn’ analysis for constitutive and stage-specific terms indicated that for all stages, a higher percentage of nodes exhibit constitutive pattern with an exception of SII. Interestingly, these constitutive nodes were identified as well-established hallmarks of ﻿cancer [36].

Although several stage-specific nodes are not well-established hallmarks of cancer, they may support the constitutive nodes to promote tumourigenesis. In fact, we observed that SII enrichment for biological processes consists of nodes that represent neural development and differentiation (Fig. S4a2), all of which have been previously implicated in not only cancer metastasis but also cancer initiation itself [37–39]. Furthermore, we observed that cellular components of SII enrichment (Fig. S4b2) concur with the expression-dependent enrichment for biological processes indicating a change in cellular projection and organization. Also, SIII enrichment for cellular components implicates the presence of genes associated with presynaptic active zone membrane (Fig. S4b3) which along with biological processes including inflammatory response and leukocyte differentiation (Fig. S4a3) have been proven to play a pivotal role in aggravating the tumour microenvironment [39].

Given the diverse pattern of enriched nodes, we explored the extent of pleiotropy of a given stage for each module by curating the genes that overlap the constitutive and stage-specific nodes (Table 3). These genes were divided by the number of correlated nodes to obtain ‘Degree of Pleiotropy’ (Table 4). Of all ESCA stages, SII indicated highest average degree of pleiotropy which along with a high degree of differentiation (Tables 2, 3 and 4) and cellular reprogramming confirms a unique tumour landscape of SII.

**Table 3:** Table indicating the number of genes that are potentially pleiotropic with their respective number of nodes and the corresponding degree of pleiotropy for each module. For number of nodes and the respective genes, the entry across the row indicates the number of genes belonging to more than one enrichment node (“constitutive” and stage-specific) with the number of nodes within the brackets. ESCA; Oesophageal Carcinoma, GO_BP; Gene Ontology Biological Processes, GO_CC; Gene Ontology Cellular Components, GO_MF; Gene Ontology Molecular Function and KEGG; Kyoto Encyclopaedia of Genes and Genomes

**ShinyGO Enrichment Module**	**Number of Potential Pleiotropic Genes with their corresponding Number of Nodes**			
	**Stage I**	**Stage II**	**Stage III**	**Stage IV**
**GO_BP**	16 (9 Nodes)	80 (24 Nodes)	29 (17 Nodes)	32 (17 Nodes)
**GO_CC**	5 (7 Nodes)	37 (21 Nodes)	8 (11 Nodes)	17 (15 Nodes)
**GO_MF**	7 (4 Nodes)	41 (18 Nodes)	8 (4 Nodes)	9 (5 Nodes)
**KEGG**	13 (10 Nodes)	11 (10 Nodes)	-	9 (7 Nodes)
**Hallmark**	4 (4 Nodes)	6 (5 Nodes)	-	7 (6 Nodes)
**WikiPathways**	12 (17 Nodes)	19 (10 Nodes)	7 (10 Nodes)	17 (29 Nodes)

**Table 4:** Table indicating degree of pleiotropy across ESCA. For each column, the entry across the row indicates the degree of pleiotropy for a given enrichment module. ESCA; Oesophageal Carcinoma, GO_BP; Gene Ontology Biological Processes, GO_CC; Gene Ontology Cellular Components, GO_MF; Gene Ontology Molecular Function and KEGG; Kyoto Encyclopaedia of Genes and Genomes

**ShinyGO Enrichment**	**Degree of Pleiotropy**			
	**Stage I**	**Stage II**	**Stage III**	**Stage IV**
**GO_BP**	1.777	3.333	1.750	1.882
**GO_CC**	0.714	1.761	0.727	1.133
**GO_MF**	1.750	2.277	2.000	1.800
**KEGG**	1.300	1.100	-	1.285
**Hallmark**	1.000	1.200	-	1.166
**WikiPathways**	0.705	1.900	0.700	0.586
**Average Pleiotropic Degree**	1.207	1.928	1.283	1.309

### Network analysis indicates robust gene regulatory network for stage II ESCA

While we identified PiGs and DaCGs that regulate ESCA-TL, it was imperative that we identify the regulatory role of DaPs via PiGs and DaCGs. Thus, we used ‘Bayesian network via splitting average’ (BNSA) strategy to identify gene regulatory networks (GRNs) specific to each stage. Further, all gene associations for a given GRN were filtered using Spearman and Pearson correlation coefficient of gene expression z-scores to identify significant associations (Table S5).

**Fig. 4:**
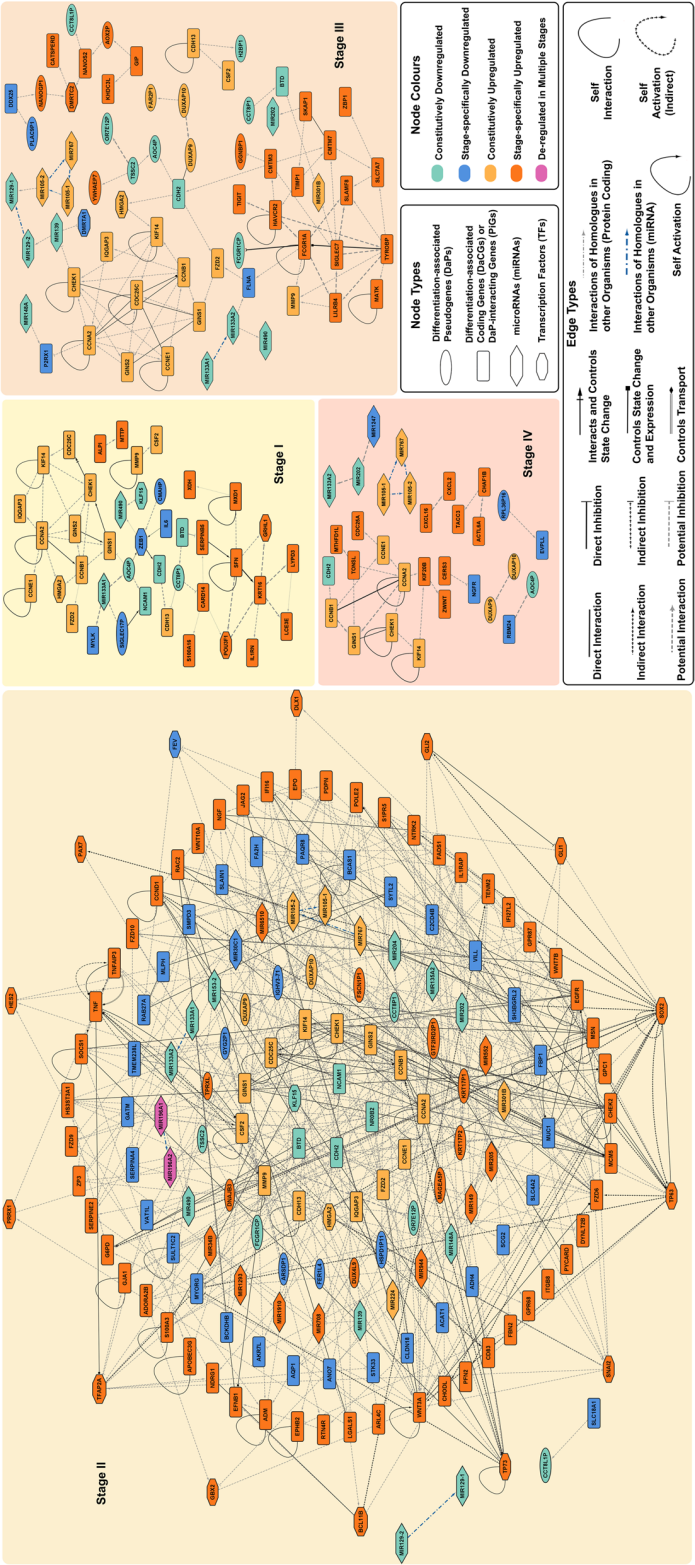
GRNs indicating the association between genes for all stages of ESCA Gene regulatory networks indicate the interactions between DaPs, PiGs, DaCGs, and miRNAs. The node types and their respective colours within a given stage are indicated in the bottom-right legend. Black edges are evidence-based interactions curated from studies that have validated these interactions experimentally, while grey edges indicate potential interactions. All interactions have a significant *p*-value for either Spearman or Pearson correlation coefficient, or both. Thin edges represent a significant *p*-value for only one correlation coefficient. Denser edges represent a significant *p*-value for both correlation coefficients, where the density is moderate for absolute values of coefficients < 0.89 and highest for absolute values of ≥ 0.89 coefficient. GRNs; Gene regulatory network, DaPs; Differentiation-associated pseudogenes, PiGs; DaP-interacting genes, DaCGs; Differentiation-associated coding genes, TFs; Transcription factors and ESCA; Oesophageal Carcinoma

We identified an intricate SII-associated GRN with a higher number of associations as compared to that of other stages (Fig. 4a). However, given that the number of genes associated with SII ESCA was higher for the aforementioned analyses, we compared the GRNs of all stages in terms of network density, where network density is inversely proportional to robustness. Robustness, defined as the ability of genes to rearrange and modulate their interactions has been previously described as a valid network parameter for topology analysis [40]. We observed that, indeed SII GRN exhibited the lowest density (Table S6). To further identify if the network topology changes with respect to the genes classified as having high degree of differentiation and/or pleiotropy, we filtered SII GRN to exhibit associated interactions that represent the same where DD is for Degree of Differentiation (Fig. S5(a)) and DP is for Degree of Pleiotropy (Fig. S5(b)). Remarkably, both DD and DP GRNs of SII displayed the lowest density, indicating that the genes belonging to SII have the capability to modulate their interactions. Furthermore, the increase in the network density for ON, DD and DP networks of SII is apparent when the DaPs are removed (Table S6). This indicates that the interaction of PiGs and DaCGs with DaPs is essential for a robust SII GRN.

Additionally, to identify key DaPs associated with each GRN, we computed local-network parameters including subgraph, degree, eigenvector, betweenness and closeness centralities. Based on the computed centralities and their respective rankings we identified that SI, II and III indicate the presence of DaPs as one of the key nodes for GRN regulation (Table S6). *AOC4P* is associated with SI and III, while *TSSC2* and *DUXAP9* are associated with SIII alone. For SII, *KRT17P2*, *KRT17P1* and *GTF2IRD2P1* were identified as key nodes that have either primary or secondary associations with transcription factors (TFs) and miRNAs that are well-known sentinels of gene regulation (Fig. 4a).

### Transcription factors and miRNAs are key regulatory elements downstream of DaPs in stage II ESCA

A previously reported evidence suggests that a unique set of TFs can trigger cellular reprogramming of fibroblasts, thus, giving rise to cells with progenitor fate belonging to the liver [41]. These reprogrammed cells when exposed to optimal oncogenic drivers underwent transformation with gene expression signatures similar to that of liver cancer cells. Since, we identified SII ESCA to be relatively enriched with TFs (Fig. 4a), especially *SOX2*, a TF previously implicated for regulating stem cell potential in a heterogeneous model of oesophageal basal cells [1], we hypothesised that TFs might trigger cellular reprogramming leading to oncogenic transformation.

**Fig. 5:**
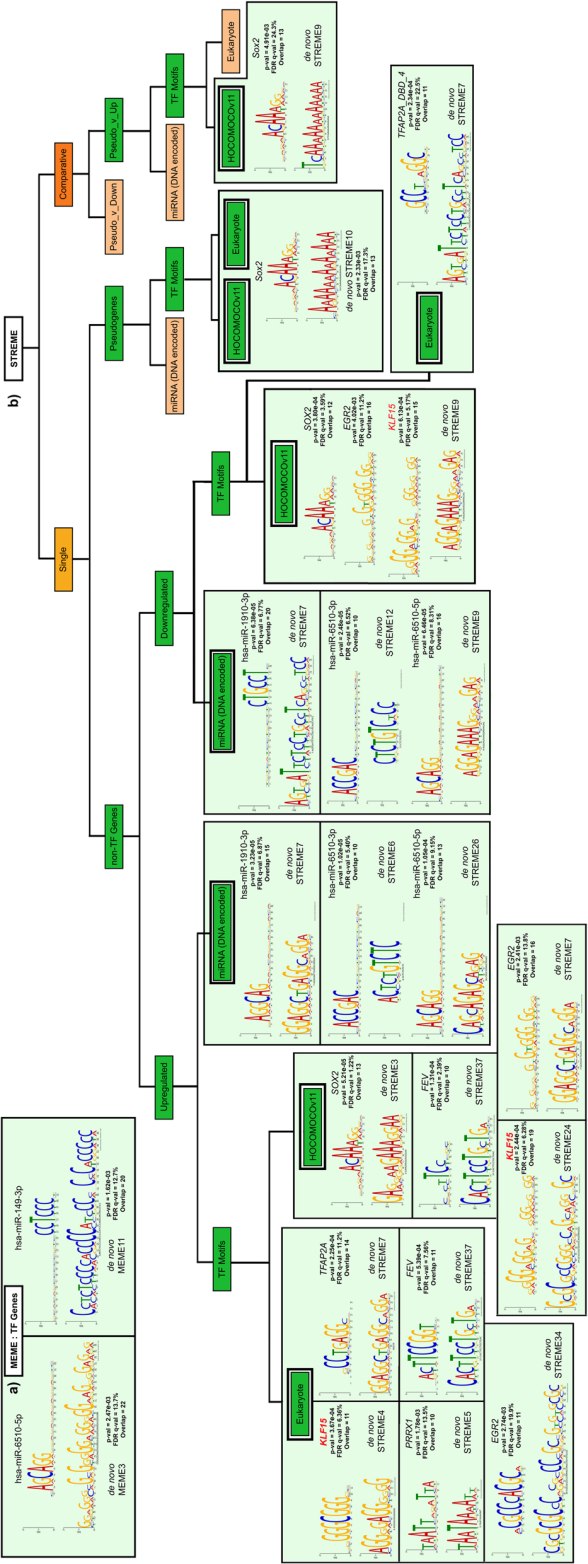
Stage II-specific Transcription Factors and microRNAs may act as Regulatory Elements Downstream of Pseudogenes Schematic of the motif analysis pipeline, consolidating *de novo* transcription factor (TF) motifs and DNA-encoded miRNA motifs obtained using MEME for TF Genes (**a**) and STREME for DaPs and non-TF Genes (**b**). For each category, the sections highlighted in green indicate the presence of *de novo* motifs with significant *p-values* and *E-values*, while the absence of the same is highlighted in faded pink. Exclusively, *de novo* motifs exhibiting a match to previously known motifs obtained via ‘Tomtom’ that correspond to Stage II-TFs and miRNAs are indicated in the bound boxes. Their respective *p-values* and FDR q-values are indicated along with gene names where genes or miRNAs in black are Stage II-specific while genes in red exhibit a “constitutive” pattern. TF; Transcription Factor, MEME; Multiple Em for Motif Elicitation, STREME; Sensitive, Thorough, Rapid, Enriched Motif Elicitation, DaPs; Differentiation-associated pseudogenes and HOCOMOCO, HOmo sapiens COmprehensive MOdel COllection

To identify such TFs, we employed ‘Simple, Thorough, Rapid, Enriched Motif Elicitation’ (STREME) followed by Tomtom for SII DaCGs and PiGs that exhibit interactions with TFs (Fig. 4a). This approach helped us identify several *de novo* TF motifs that have significant overlap with *SOX2, PRRX1, TFAP2A* and *FEV* (Fig. 5b). Furthermore, STREME37 is enriched in upregulated DaCGs alone which overlaps with *FEV*, a downregulated TF, thus, confirming its role as a transcriptional repressor. Similarly, *PRRX1* may act as a transcriptional activator while *SOX2* and *TFAP2A* act as both activators and repressors. Interestingly, we also identified enrichment of *KLF15*, a constitutively expressed TF motif and *EGR2*, a motif identified as a part of differential expression analysis but not in SII GRN.

While we identified, SII-specific *de novo* TF motifs, we wanted to understand their role as effectors of cellular reprogramming. Thus, we employed STREME for absolute as well as relative enrichment (Fig. 5b) of TF motifs in DaPs. Interestingly, only *SOX2* had significant overlap with the *de novo* motifs in both individual as well as relative enrichment (STREME10 and STREME9, respectively). Further analysis using Motif Alignment and Search Tool (MAST) indicated that STREME9 (Fig. 5b) is relatively more enriched in SII-specific DaPs including, *ARSDP1*, *FER1L4*, *TPRXL* and *GTF2IRD2P1* (Fig. S6a-f, i, l-o) as opposed to a single DaCG, *TENM2* (Fig. S6p). This suggests that *SOX2* not only acts as a TF upstream of DaPs for cellular reprogramming, but it may also act downstream of DaPs along with *PRRX1*, *TFAP2A* and *FEV* for promoting oncogenic transformation.

The primary mode of regulatory function of pseudogenes is by acting as competing endogenous RNAs (ceRNAs) for miRNAs against functional mRNAs as a part of post-translational regulation [13]. However, miRNAs have also been studied for their involvement in transcriptional regulation [42]. Hence, we explored the possibility of miRNAs as transcriptional regulators using MEME and STREME on DaPs, TF and non-TF genes. Indeed, our MEME analysis revealed that SII-specific *de novo* miRNA-motifs (*MIR149* and *MIR6510*) are enriched in TF genes (Fig. 5a), suggesting that DaPs may transcriptionally regulate *SOX2*. Furthermore, known miRNA motifs using Tomtom on *de novo* motifs (STREME) also revealed potential transcriptional regulation of DaCGs via *MIR6510* and *MIR1910*. Our results thus, indicate that DaPs may exhibit a pleiotropic role through transcriptional regulation of downstream TFs and other genes through miRNA pool regulation.

### Unique combination of APOBEC gene dysregulation promotes Hypermutation in SII ESCA

Complex mutational landscape, a chief cancer hallmark is frequently associated with genes belonging to the APOBEC (apolipoprotein B mRNA editing enzyme, catalytic polypeptide-like) superfamily due to their function as an RNA editing enzyme [43, 44]. While we identified *APOBEC3G* (A3G) dysregulation in SII ESCA, its role in imparting significant somatic hypermutations is currently unknown as A3G has been traditionally studied for its role in either providing immunity against a plethora of viruses or regulating tumour immune landscape [43, 45, 46]. Indeed, our analysis does indicate potential immune-related A3G activity due to its primary association with *SOCS1* and secondary associations with *TNF, TNFAIP3, TP73, MUC1* and *EGFR* (Fig. 4a-b)*.* However, its unique sequence preference for CC-nucleotide and stage-specific dysregulation prompted us to investigate stage II mutational landscape (SII-ML) in the context of A3G.

The presence of localised regions of somatic hypermutations (Kataegis) reflects APOBEC gene dysregulation, hence, we explored all stages of ESCA for kataegis using MAF tools [47]. While kataegis was observed across chromosomes 1-2, 7, 9 and 17-19 (Fig. S7a), stage-specific analysis revealed that only SII and SIII showed significant events of kataegis with SII indicating higher hypermutations (Fig. S7b). Since SIII ESCA displayed kataegis, we further analysed the distribution of other dysregulated APOBEC genes. Indeed, *APOBEC3A* (A3A) was upregulated across all stages while *APOBEC3B* (A3B) was upregulated in all except SIV. Despite dysregulation of APOBEC genes, SI and SIV showed no signs of kataegis suggesting that A3G along with other de-regulated APOBEC genes may impart significant tumour mutational burden for SII.

### Combinatorial and non-combinatorial expression of DaPs results in diverse mutational landscape and degree of differentiation

To understand if a differential mutation pattern exists with respect to the expression of DaPs, we analysed SII in the context of the combined expression pattern of all SII DaPs. The assumption for this approach is that the expression pattern and associated GRN can be largely extrapolated to patients diagnosed with SII ESCA with only a few exceptions owing to stochastic events. Thus, we divided the patient samples into two different cohorts based on their expression pattern of DaPs; UDaP_Combo to indicate ‘Upregulated & downregulated DaPs Combination’ and vice versa.

**Fig. 6:**
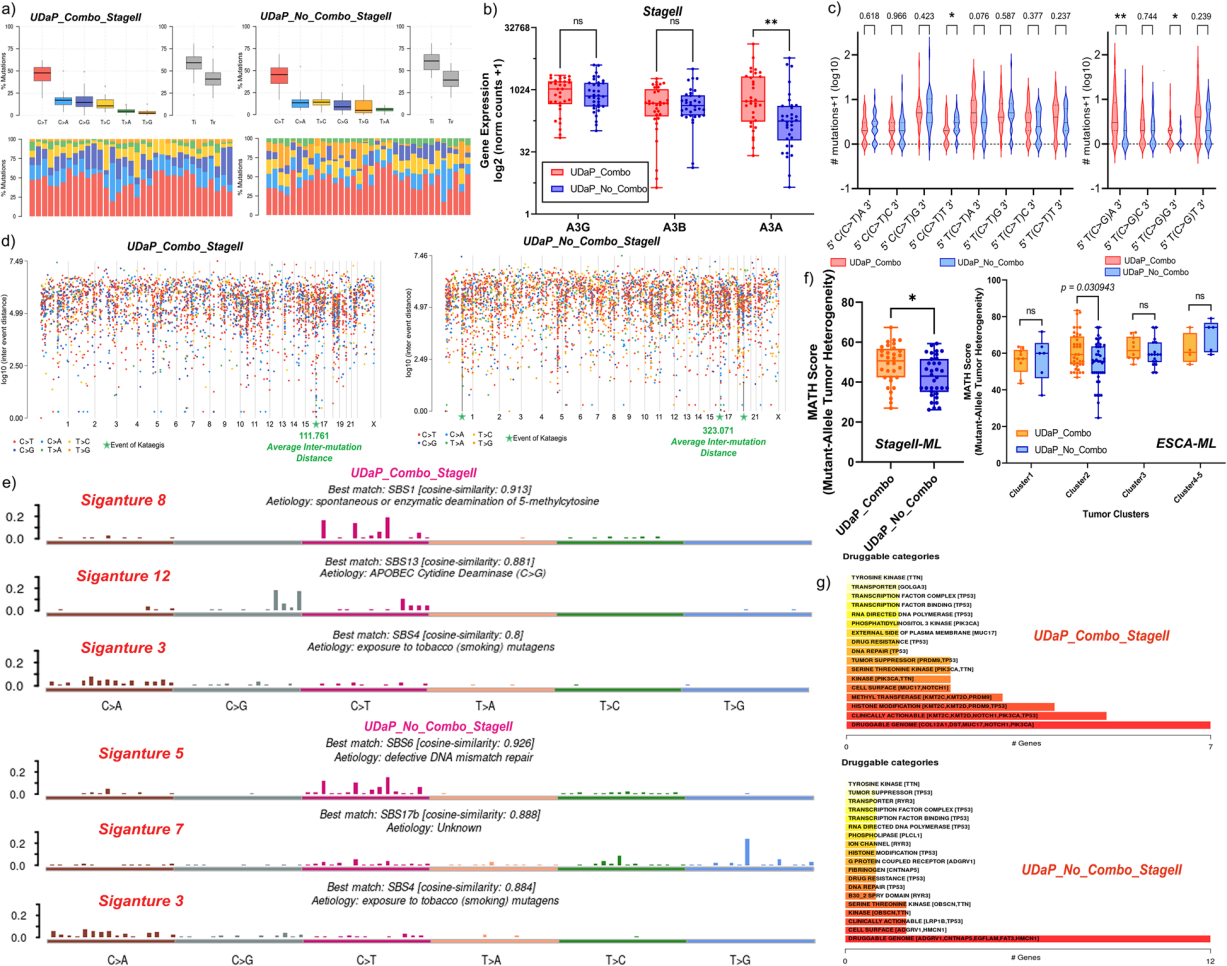
Combinatorial Dysregulation of DaPs promote Unique Mutation Landscape with High Tumour Heterogeneity (**a**) Stacked bar plot indicating the fraction of six classes of single base substitutions within a given sample for UDaP_Combo and UDaP_No_Combo of stage II ESCA. Box plot indicating the total percentage of single base substitutions across all samples of a given cohort (top left) along with a box plot indicating the percentage of transitions (Ti) and transversions (Tv) (top right). **b** Box and whisker plot indicating the expression of *APOBEC3G* (A3G), *APOBEC3B* (A3B) and *APOBEC3A* (A3A) for UDaP_Combo and UDaP_No_Combo. Significance of expression between the two groups was evaluated using Mann-Whitney tests, indicated using asterisks above the boxplot as **p < 0.01 and ns for non-significant (**c**) Violin Plot indicating the distribution of mutations associated with A3G and A3A motifs for C-base transitions (left), and A3A motifs for C-base transversions (right). The significance of expression between the two groups was evaluated using Mann-Whitney tests, indicated using asterisks above the boxplot as **p < 0.01, **p < 0.05 and non-significance is indicated by their respective *p*-values. **d** Rainfall plot indicating the events of kataegis across chromosomes 1-22 and X (x-axis). Kataegis is represented by a green star along with the average inter-mutation distance as obtained from MAF tools. The class of single-base substitutions that constitute the hypermutations are represented below the plot (**e**) Bar plot indicating the single base mutation signatures (SBS) associated with UDaP_Combo (top) and UDaP_No_Combo (bottom) along with their best match for validated signatures and their respective aetiology. **f** Box and whisker plot indicating the MATH scores of UDaP_Combo and UDaP_No_Combo of stage II ESCA (left), and cluster-wise MATH scores across all stages of ESCA (right). **g** Bar plot indicating potential categories of druggable genes and the top 5 genes associated with the categories for UDaP_Combo (top) and UDaP_No_Combo (bottom)

The MAF files were segregated accordingly for both cohorts and subject to mutation analysis as described before [26]. Frequency distribution of base substitution classes indicated that while cumulative APOBEC mutations (C>T and C>G) are favoured for both cohorts (Fig. 6a), T>C (non-APOBEC) is favoured instead of C>G in UDaP_No_Combo. Thus, we hypothesised that the difference in expression of A3A/B/G between the two cohorts results in higher C>G preference in UDaP_Combo. As expected, A3A upregulation was significantly higher in UDaP_Combo which concurs with a higher C>G preference (Fig. 6b). We further investigated the distribution of APOBEC mutation motifs (tCw for A3A/B and cCw for A3G) to confirm the sequence preference. Indeed, tCw motifs (5ʹ-T(C>G)A-3ʹ and 5ʹ-T(C>G)G-3ʹ) were favoured in UDaP_Combo while, cCw motifs (5ʹ-C(C>T)T-3ʹ) were favoured in UDaP_No_Combo (Fig. 6c). It was interesting to note that despite A3A upregulation being significantly higher in UDaP_Combo, there were lower hypermutations with average inter-mutation distance (A-IMD) being close to that of high-A3G enriched samples and low-A3A enriched samples (Fig. 6d and Fig. S7c). This may suggest that the variables A-IMD, hypermutations (number) and APOBEC gene expression are independent.

Further analysis of single base signatures (SBS) of mutations based on the cophenetic metric (Fig. S7d) revealed that UDaP_Combo has higher signatures (SBS1 and SBS13) of enzymatic deaminase activity (Fig. 6e) which is in accordance with our previous results where A3A-associated substitutions were favoured in UDaP_Combo. Interestingly, while SBS13 is attributed to a tumour mutation signature, it has also been regarded as a homeostatic mutation signature of the small intestine [48]. In fact, GSEA analysis revealed that UDaP_Combo has a higher number of intestine-associated cell signatures negatively enriched for normal samples. Furthermore, LEA-GSEA for both cohorts revealed that both average as well as individual enriched degree of differentiation are higher for UDaP_Combo (Table S4).

We further calculated the Mutant-Allele Tumour Heterogeneity (MATH) Score [49] for both cohorts, where a higher MATH score indicates the presence of more clones giving rise to higher tumour heterogeneity which may lead to a high rate of metastasis and disease recurrence. Indeed, the characteristic mutations within the UDaP_Combo showed significantly higher MATH score (Fig. 6f). Further analysis of cluster distribution over ESCA revealed that tumours with cluster2 heterogeneity alone were significantly different where a higher fraction of cluster2 samples belonged to that of SII ESCA. We further analysed the difference in druggability between the two cohorts and identified that UDaP_Combo had a lesser number of druggable targets as well as categories (Fig. 6g). This indicates that combinatorial expression of DaPs along with A3A/G characterises the mutation landscape with higher clonal heterogeneity to potentially promote metastasis and disease recurrence.

### Diverse gene expression dependent poor prognosis associated with combinatorial and non-combinatorial dysregulation of DaPs

**Fig. 7:**
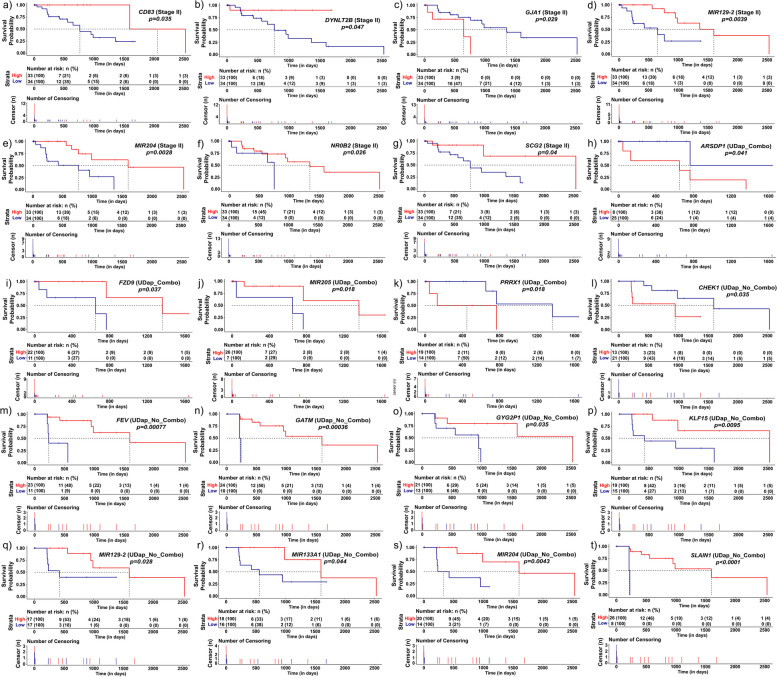
Distinct DaPs, TFs, miRNAs and DaCGs are associated with Poor Prognosis for Combinatorial and Non-combinatorial Dysregulation of DaPs in SII ESCA Kaplan-Meier plots indicating survival probability over increasing time for a-g) SII with respect to expression of *CD83* (**a**), *DYNLT2B* (**b**), *GJA1* (**c**), *MIR129-2* (**d**), *MIR204* (**e**), *NR0B2* (**f**), and *SCG2* (**g**), **h**-**k**) UDaP_Combo with respect to expression of *ARSDP1* (**h**), *FZD9* (**i**), *MIR205* (**j**) and *PRRX1* (**k**), and **l**-**t**) UDaP_No_Combo with respect to expression of *CHEK1* (l), *FEV* (**m**), *GATM* (**n**), *GYG2P1* (**o**), *KLF15* (**p**), *MIR129-2* **(q**), *MIR133A1* (**r**), *MIR204* (**s**) and *SLAIN1* (**t**). The tables below each graph indicate risk table (top) and censored population table (bottom). The number of patients at risk is indicated in numbers with the respective percentages in the bracket for risk table. The censored observations for each variable are indicated by their respective colour for censored population table. ESCA; Oesophageal Carcinoma, SII; Stage II, UDaP_Combo; Upregulated & downregulated DaPs Combination and UDaP_No_Combo; Upregulated & downregulated DaPs No Combination

Unique mutational landscape and degree of differentiation prompted us to hypothesise that distinct genes affect both cohorts in terms of survival. As expected, univariate cox regression analysis revealed that distinct genes are associated with poor survival with a higher number of genes in UDaP_No_Combo [9] as compared to that of UDaP_Combo [4] (Table 5). For DaPs, above-median expression of *ARSDP1* was associated with UDaP_Combo (Fig. 7h) and vice versa for *GYG2P1* in UDaP_No_Combo (Fig. 7o). Similarly, among TFs, high *PRRX1* promoted poor survival within UDaP_Combo (Fig. 7k) and low *FEV* within UDaP_No_Combo (Fig. 7m). Interestingly, *KLF15* despite being a constitutively de-regulated TF, was only associated with poor prognosis for UDaP_No_Combo for downregulation (Fig. 7p). Among the DaCGs, a pattern similar to that of *KLF15* was observed for *CHEK1* in UDaP_No_Combo (Fig. 7. l) despite being constitutively upregulated. Other DaCGs that are associated with poor survival within UDaP_No_Combo include low *GATM* and low *SLAIN1* (Fig. 7n and t).

**Table 5:** Table indicating the hazard's ratio obtained from coxph regression for UDaP_Combo and UDaP_No_Combo. N represents the number of samples with above median (high) or below median (low) gene expression as the variable. For hazard's ratio (HR), <1 indicates low risk and >1 indicates high risk to survival with respect to the reference for a given variable. The 95% confidence intervals for HR are represented in the bracket along with Likelihood Ratio (LR) and Wald’s Test (W) *p-value*, where *p-values* ≤ 0.05 are highlighted in bold italic

	**Univariate**						**Multivariate**		
	**Variable**		**N**	**Hazard's Ratio**	***p-value (W)***	***p-value (LR)***	**Hazard's Ratio**	***p-value (W)***	***p-value (LR)***
**UDaP_Combo**	*ARSDP1*	High	8	Reference	-	***0.040***	Reference	-	0.080
		Low	25	0.14 (0.016-1.2)	0.077		0.29 (0.025-3.4)	0.323	
	*FZD9*	High	22	Reference	-	***0.040***	-	-	
		Low	11	7.5 (0.82-68)	0.074		-	-	
	*MIR205*	High	26	Reference	-	***0.030***	Reference	-	
		Low	7	9.7 (0.99-96)	0.051		2.41 (0.151-38.4)	0.534	
	*PRRX1*	High	19	Reference	-	***0.030***	Reference	-	
		Low	14	0.1 (0.011-1)	0.051		0.29 (0.021-3.9)	0.350	
**UDaP_No_Combo**	*CHEK1*	High	13	Reference	-	***0.050***	Reference	-	***0.002***
		Low	21	0.23 (0.053-1)	***0.050***		5.14 (0.228-116)	0.303	
	*FEV*	High	23	Reference	-	***0.005***	Reference	-	
		Low	11	12 (2-67)	***0.006***		20.24 (1.308-313.1)	***0.031***	
	*GATM*	High	24	Reference	-	***0.020***	Reference	-	
		Low	10	15 (2.1-109)	***0.007***		0.29 (0.013-6.7)	0.441	
	*GYG2P1*	High	21	Reference	-	***0.040***	Reference	-	
		Low	13	5 (0.97-26)	0.054		7.74 (0.494-121.3)	0.145	
	*KLF15*	High	19	Reference	-	***0.010***	Reference	-	
		Low	15	6.2 (1.3-30)	***0.023***		58.59 (1.160-2958.1)	***0.042***	
	*MIR129-2*	High	17	Reference	-	***0.040***	-	-	
		Low	17	7.5 (0.82-68)	***0.042***		-	***-***	
	*MIR133A1*	High	18	Reference	-	***0.040***	Reference	-	
		Low	16	4.5 (0.91-22)	0.064		6.82 (0.209-222.4)	0.28	
	*MIR204*	High	20	Reference	-	***0.007***	-	-	
		Low	14	7.5 (1.5-38)	***0.014***		-	***-***	
	*SLAIN*	High	26	Reference	-	***0.007***	-	-	
		Low	8	30 (2.7-341)	***0.006***		-	***-***

We also observed distinct miRNAs associated with poor survival between the two cohorts. While low *MIR205* is associated with UDaP_Combo (Fig. 7j), below median expression of *MIR129-2, MIR133A1* and *MIR204* led to poor prognosis in UDaP_No_Combo (Fig. 7q-s). Furthermore, while the expression pattern related to poor survival was in accordance with the pattern observed across SII ESCA (Fig. 4a), the prognosis was specific to individual cohorts. It was interesting to note that while we observed overexpression of *FZD9* in SII ESCA, below median expression of *FZD9* was associated with poor survival in UDaP_Combo (Fig. 7i). We further explored if a similar pattern of expression-dependent prognosis was evident across the entire SII landscape. We observed that none of the DaPs had a significant effect on survival across the overall landscape. While dysregulation of the TFs, *PRRX1*, *FEV* and *KLF15* did not affect prognosis, *NR0B2*, a constitutively downregulated TF resulted in poor survival (Fig 7f). Similarly, we observed only *CD83* (low), *DYNLT2B* (low), *GJA1* (high) and *SCG2* (low) were associated with poor survival (Fig. 7a-c, g). Interestingly, only two miRNAs had a significant overlap of poor prognosis across UDaP_No_Combo as well as SII ESCA (Fig. 7d-e).

Multivariate cox regression analysis revealed that all the aforementioned genes except *FEV* and *KLF15* were independent variables of survival (Table 5). Low expression of both *FEV* and *KLF15* was associated with poor survival while higher expression of both TFs was associated with prolonged survival (Fig. S8b). Further analysis of stage-dependent survival indicated that despite having a higher degree of differentiation, pleiotropy and a unique mutation landscape, SII did not exhibit poor survival as compared to that of SIII and IV (Fig. S8a).

Interestingly, patient stratified survival analysis for SII and ≥ SIII tumours revealed a favourable prognosis for UDaP_No_Combo only with respect to ≥ Stage III tumours, but not UDaP_Combo (Fig. S8c and Table S7). This suggests that Stage II UDaP_Combo is more likely an intermediate stage that has a high probability of transforming into Stage III tumours. Also, however, given that they have a high degree of pleiotropy, therapeutic intervention can potentially help UDaP_Combo to undergo molecular changes to mimic the tumour microenvironment of UDaP_No_Combo with better overall survival. Hence, SII presents itself as an early stage with better overall survival, however, patient stratification and their respective analysis prompt its potential as a highly unstable stage with though not equal, but indistinguishable prognostic events with respect to that of SIII.

## Discussion

Cancer estimates by GLOBOCAN denote oesophageal carcinoma as the 6^th^ leading cause of cancer deaths [50]. Furthermore, while the cancer incidences for ESCA decreased as compared to that of 2018, cancer mortality increased by 0.2% as of 2020 [50, 51]. An apparent increase in mortality despite a decrease in incidences implies the lack of appropriate biomarkers for early diagnosis and precise drug treatment options. Furthermore, the presence of metaplastic epithelium signifies the need to characterise the unexplored heterogenous events of each stage of ESCA [3, 4, 6, 7].

Albeit unconventional, non-coding RNAs can aid in the understanding of heterogeneous cellular events associated with cancer and its progression [13–15]. In fact, our analysis highlights not only the unique expression pattern of DaPs but also their interactions with PiGs in potentially regulating tumour progression. For instance, in SI, *SIGLEC17P (SP)* and its PiG, *NCAM1 (N1)* are both downregulated and exhibit a significant positive correlation. NCAM1 expression is positively associated with cancer-associated neurogenesis and negatively with tumour invasiveness [38, 52]. Thus, SP-N1 interaction may promote invasiveness in SI ESCA. However, a significantly better overall survival prompts a potential dual role of SP-N1 in limiting neurogenesis as well regulating the invasiveness in SI ESCA. Similarly, in SII, *DNAJB3 (D3)* and its PiG *KIF14 (K14)* are both upregulated and exhibit a positive correlation. High-throughput experimental evidence suggests that D3-K14 interaction is a part of the midbody interactome that plays a significant role in regulating cytokinesis. However, *KIF14* also regulates gastric cancer progression and metastasis via p-Akt [53]. This suggests that while constitutive dysregulation of *KIF14* regulates p-Akt across all stages, D3-K4 interaction may additionally have a significant role in regulating cytokinesis and associated events during differentiation and cellular reprogramming. However, since both SP-N1 and D3-K14 interactions are uncharacterized [54, 55], their role in regulating aforementioned events within ESCA warrants experimental investigation.

We define unconventional yet an effective method to estimate differentiation and cellular reprogramming. In fact, our proposed metrics of degree of differentiation and cellular reprogramming aided in the quantification of the extent of the aberrant events of turnover that have been previously established as key factors of pre-cancerous events of ESCA associated with Barret’s oesophagus (BE) [2, 3]. Furthermore, the near-identical degree of differentiation between BE and SII helped us validate the potential of BE to transform into SII. Also, a high rate of cellular reprogramming and associated differentiation may in turn indicate the need for relatively rapid change in the interactions between genes to modulate their functionality which in turn represents the robustness of a given gene regulatory network [40]. Indeed, stage II ESCA with a high degree of BE-type differentiation and cellular reprogramming was identified to be the most robust network that promotes a higher capacity to modulate gene-gene interactions demonstrating a shift towards oncogenic transformation.

While cellular reprogramming followed by oncogenic transformation was revealed as a valid model to understand the seeding events of hepatocellular carcinoma using fibroblasts [41], an identical model to understand the transforming events of oesophageal carcinoma is currently not available. Our analysis, however, reveals the key combination of transcription factors (TFs) including *FEV*, *TFAP2A*, *PRRX1* and *SOX2* that we believe can be used to establish a similar model for cellular reprogramming induced oncogenesis in oesophageal cells. In fact, *SOX2* has previously been reported as a regulator of stem cell potential for oesophageal basal cells [1] and thus, can be regarded as a critical component for the cellular reprogramming in oesophageal cells that may lead to oesophageal carcinoma. Furthermore, unconventional patient-stratification of stage II with respect to combinatorial dysregulation of DaPs followed by survival analysis identified *FEV* and *PRRX1* as key transcription factors associated with poor prognosis while *SOX2* had no significant effect in both cohorts. This may indicate that *SOX2* acts as an effector of cellular reprogramming while *FEV* and *PRRX1* are more inclined towards regulating the oncogenic transformation in the distinct landscape of pseudogene expression. However, given the stage-specific dysregulation of the aforementioned TFs, it is imperative that further stage-specific probing is needed to provide a robust model of reprogramming-induced oncogenic transformation in ESCA.

Remarkably, DaP dysregulation-based sample stratification and further analysis of its degree of BE-type differentiation signified the importance of DaPs and their dysregulation in a specific combination as a hallmark of increased transdifferentiation and transcommitment leading to diverse GI^Non-oesophageal^ as well as lung CSigns. The relatively higher extent of differentiation may further promote aberrant events of mutation, a well-known hallmark of metaplastic epithelium that leads to ESCA [6, 7]. Indeed, we observed a relatively higher number of genes associated with the APOBEC family (cytidine deaminases that promote single base substitutions) to be dysregulated across stage II ESCA with A3A having significantly higher expression across combinatorial dysregulation of DaPs as compared to that of other samples. Thus, indicating a potential correlation between combinatorial dysregulation of DaPs, degree of differentiation and APOBEC-associated mutations.

Interestingly, APOBEC mutagenesis is also regarded as a homeostatic event of the small intestine [48]. In fact, our analysis of single base substitution across both cohorts revealed relatively higher A3A-associated substitutions for combinatorial dysregulation of DaPs which along with a higher enrichment of small-intestine associated cell signatures suggest A3A as a driver of the homeostatic mutagenic events of reprogrammed oesophageal cells. However, a comparative analysis between normal as well as cancerous tissues of the intestine and oesophagus is required as conclusive evidence to implicate A3A as a promising marker of homeostatic mutagenic events within reprogrammed oesophageal cells. In conclusion, through our current study, we present a unique pipeline presenting three unconventional yet valid metrics to measure and elucidate cellular reprogramming and oncogenic transformation using differentiation-associated coding and non-coding genes that present a specific set of dysregulated DaPs and TFs as markers of early-stage ESCA. Additionally, these metrics aided in distinct patient-stratification that implicate DaPs as regulators of differentiation that promote indistinguishable prognostic events between early and late-stage ESCA.

### Supplementary Information

**Additional file 1:** **Figure S1.** Subset of DaPs exhibit Stage-specific expression pattern across ESCA Box and whisker plots indicating the gene expression profile of DaPs differentially expressed in Stage I (a), Stage II (c), Stage III (e) and Stage IV (g) ESCA as compared to that of normal. Significance of expression between the two groups for each DaP was evaluated using Mann-Whitney tests; indicated by asterisks above the boxplot as *p < 0.05, **p < 0.01 and ns (non-significant). Volcano plots of differentially expressed genes in Stage I (b), Stage II (d), Stage III (f) and Stage IV (h) ESCA indicating down-regulated and up-regulated DaPs shown as green and red dots, respectively. The cut-off for |log2 FC| ≥ 1.5 is indicated by dashed-lines along x-axis, while the dashed-line along y-axis indicates cut-off for -log10(adjusted *p*-value) > 1.30103. Additionally, stage-specific downregulation of DaPs is indicated by orange dots, while pale-green dots represent stage-specific upregulation. Genes with adjusted *p*-value <-log10(0.05) and/or |log2FC| < 1.5 are indicated by grey dots. DaPs; Differentiation-associated pseudogenes, FC; Fold Change and ESCA; Oesophageal Carcinoma.

**Additional file 2:** **Figure S2.** PiGs, DaGs and miRNAs exhibit unique expression pattern similar to that of DaPs across ESCA a-l) Volcano plots associated with Stage I (a-c), Stage II (d-f), Stage III (g-i) and Stage IV (j-l) ESCA indicating differentially expressed PiGs (a, d, g and j for Stage I, II, III and IV, respectively), DaGs (b, e, h and k for Stage I, II, III and IV, respectively) and miRNAs (c, f, i and l for Stage I, II, III and IV, respectively). PiGs and their interactions with DaPs across each stage are labelled as ‘PiG-DaP’ combination for their respective dots using a line. Green dots indicate down-regulation, while red dots indicate up-regulation for PiGs and DaCGs. Similarly, for miRNAs, downregulation is indicated by green diamonds, while upregulation is indicated by red diamonds. Dashed-lines along x-axis indicate the cut-off for|log2 FC| ≥ 1.5, while the dashed-line along y-axis indicates cut-off for-log10(adjusted *p*-value) > 1.30103. Additionally, for PiGs and DaGs, stage-specific downregulation is indicated by orange dots, while pale-green dots represent stage-specific upregulation. Similarly, for miRNAs, orange diamonds indicate stage-specific downregulation, while pale-green diamonds indicate stage-specific upregulation. Yellow dots and diamonds indicate de-regulation across all stages of ESCA, referred to as ‘Constitutively De-regulated’ for PiGs and miRNAs, respectively. Lavender diamonds indicate miRNAs de-regulated across more than one stage, with the stages of ESCA indicated in the brackets in their respective box of legends. Genes with adjusted *p*-value <-log10(0.05) and/or |log2FC| < 1.5 are indicated by grey dots for PiGs and DaGs, while for miRNAs they are represented using grey diamonds. DaPs; Differentiation-associated pseudogenes and ESCA; Oesophageal Carcinoma.

**Additional file 3:** **Figure S3.** Distinct Biological Processes, KEGG and Immune Signatures are enriched for de-regulated PiGs and DaCGsa) LEA plots (left) indicating the correlation between the enriched biological processes for Stage I ESCA with the corresponding expression heatmap of LEA genes (right). b-c) LEA plots (left) indicating the correlation between the enriched biological processes (b) and KEGG pathways (c) for Stage II ESCA and their respective heatmaps indicating expression of LEA genes (right). d) Heatmap indicating expression of LEA genes enriched for biological processes across Stage III ESCA e) LEA plot (left) indicating the correlation between the enriched immune signatures for Stage III ESCA and respective heatmap indicating expression of LEA genes (right). (f) LEA plot (top) indicating the correlation between enriched biological processes for Stage IV ESCA with the corresponding expression of LEA genes (bottom). For all heatmaps, blue indicates downregulation while red indicates upregulation in cancer samples with respect to normal. KEGG; Kyoto Encyclopaedia of Genes and Genomes, PiGs; DaP-interacting genes, DaP; Differentiation-associated Pseudogenes, DaCGs; Differentiation-associated Coding Genes, LEA; Leading Edge Analysis and ESCA; Oesophageal Carcinoma.

**Additional file 4:** **Figure S4. **Expression-Independent Enrichment landscape of differentially expressed PiGs and LEA-DaCGs across various stages of ESCA a-f) Pie charts indicating the distribution of enriched terms obtained using ShinyGO across various stages of ESCA for biological processes (a), cellular components (b), molecular functions (c), KEGG pathways (d), hallmarks (e) and wiki-pathways (f). a1-f4) Chord diagrams indicating the association between the stage-specific and “constitutively” enriched biological processes (a1-a4), cellular components (b1-b4), molecular functions (c1-c4), KEGG pathways (d1-d3), hallmarks (e1-e3) and wiki-pathways (f1-f4). Enrichments exhibiting “constitutive” pattern are highlighted in purple while stage-specific enrichment is highlighted in black. Nodes are indicated in blue with more significantly enriched terms as dark blue and a decrease in significance indicated as a decrease in the colour. The association between nodes is represented using red edges where bright red indicates higher overlap of genes and a decrease in overlap is indicated by decrease in the colour.

**Additional file 5: FigureS5.** GRNs indicating the association between genes for all stages of ESCA Gene regulatory networks indicating the interactions between DaPs, PiGs, DaCGs and miRNAs for Degree of Differentiation or DD (a) and Degree of Pleiotropy or DP (b). DD represents interactions between DaPs, miRNAs and genes identified as part of LEA, while DP represents interactions between DaPs, miRNAs and genes having functional pleiotropy. The node types and their respective colours within a given stage are indicated in the bottom-right legend. Black edges are evidence-based interactions curated from studies that have validated these interactions experimentally, while grey edges indicate potential interactions. All interactions have a significant *p*-value for either Spearman or Pearson correlation coefficient, or both. Thin edges represent a significant *p-*value for only one correlation coefficient. Denser edges represent a significant*p*-value for both correlation coefficients, where the density is moderate for absolute value of coefficients < 0.89 and highest for absolute value of ≥ 0.89 coefficient. GRNs; Gene regulatory network, DaPs; Differentiation-associated pseudogenes, PiGs; DaP-interacting genes, DaCGs; Differentiation-associated coding genes, TFs; Transcription factors and ESCA; Oesophageal Carcinoma.

**Additional file 6: Figure S6.** *SOX2* regulates a subset of stage II-specific DaPs and DaCGs MAST results indicating motif alignment of *de novo *STREME9 motif identified from relative enrichment of motifs in DaPs as compared to that of DaCGs and PiGs having putative or known interactions with stage II TFs. STREME9 motif alignment across stage II-specific DaPs (a-f, i, l-o) and DaCGs (p) as well as constitutively de-regulated DaPs (g-h, j-k) along with their respective *p-values*, sequence alignment range and strand direction. The horizontal bar beside the gene name indicates gene sequence length (462bp for DaPs and 4000bp; truncated to 462bp for DaCGs) with the left vertical bar indicating the start of visual range of motif alignment while the right vertical bar indicates the end of visual range. The plus and minus sign alongside the sequence indicates strand direction with the red vertical bars indicating the aligned motifs. Motif on positive strand indicates normal motif, while motif on negative strand indicates reverse complement. The individual *p-values*associated with motif is indicated above the aligned motif, while the overall *p-values* are indicated along with the gene names.

**Additional file 7:** **Figure S7. **Unique Mutational Landscape of Combinatorial de-regulation of DaPsa) Rainfall plot indicating the events of kataegis (green star) across the chromosomes 1-22 and X (x-axis) for entire ESCA (a), each stage of ESCA (b) and samples stratified based on the median cut-off of APOBEC gene expression (c). Kataegis is represented by a green star along (a-c) with the average inter-mutation distance as obtained from MAF tools (c). The class of single-base substitutions that constitute the hypermutations are represented below the plot. The APOBEC genes that are upregulated are represented in lavender (a-b) with the extent of differential expression as Log2FC and respective *p-adjusted values *(b). The class of single-base substitutions that constitute the hypermutations are represented below the plot d) An elbow plot indicating the number of signatures as clusters on x-axis generated through unsupervised learning, non-negative matrix factorization. The number of signatures is decided by a significant drop in the correlation coefficient.ll plot indicating the events of kataegis (green star) across the chromosomes 1-22 and X (x-axis) for entire ESCA (a), each stage of ESCA (b) and samples stratified based on the median cut-off of APOBEC gene expression (c). Kataegis is represented by a green star along (a-c) with the average inter-mutation distance as obtained from MAF tools (c). The class of single-base substitutions that constitute the hypermutations are represented below the plot. The APOBEC genes that are upregulated are represented in lavender (a-b) with the extent of differential expression as Log2FC and respective *p-adjusted values *(b). The class of single-base substitutions that constitute the hypermutations are represented below the plot d) An elbow plot indicating the number of signatures as clusters on x-axis generated through unsupervised learning, non-negative matrix factorization. The number of signatures is decided by a significant drop in the correlation coefficient.

**Additional file 8: Figure S8.** Stage II ESCA may act as potential Tipping Point for Differentiation induced Oncogenesis Kaplan-Meier plots indicating survival probability over increasing time for a) all stages of ESCA b) varied combinatorial expression of *FEV *and *KLF15*for SII UDaP_No_Combo and c) patient stratified Stage II and tumours ≥ Stage III. The tables below each graph indicate risk table (top) and censored population over time (bottom). The number of patients at risk is indicated in numbers with the respective percentages in the bracket for risk table. The censored observations for each variable are indicated by their respective colour for censored population table. ESCA; Oesophageal Carcinoma, SII; Stage II, UDaP_Combo; Upregulated& downregulated DaPs Combination and UDaP_No_Combo; Upregulated& downregulated DaPs No Combination.

**Additional file 9: Table S1.** List of downregulated pseudogenes and their potential interactors obtained from NCBI and PubMed.

**Additional file 10: Table S2.** List of upregulated pseudogenes and their potential interactors obtained from NCBI and PubMed.

**Additional file 11: Table S3.** B) Table indicating differentially expressed genes across Stage II ESCA. Genes with constitutive expression pattern are highlighted as red cells for upregulation and green for downregulation, while lavendar cells indicate miRNAs differentially expressed in more than one stage of ESCA. Uncolored cells indicate de-regulated genes with stage-specific expression pattern. DaPs; Differentiation-associated Pseudogenes, PiGs; DaP-interacting genes, DaCGs; Differentiation-associated coding genes that have no known interactions with DaP, miRNAs; microRNAs.

**Additional file 12: Table S4.** G) Table indicating cell signatures and biological processes differentially  enriched between Stage III EAC and Normal, sorted by Normalized Enrichment Score (NES). Green cells  (with associated GSEA Ids in bracket) indicate potential events of transdifferentiation and transcommitment leading to cell signatures that represent adjacent tissues, while grey cells indicate events associated with the resident tissue. Lavendar cells indicate potential events of cellular reprogramming of cells that exhibit characteristics of respiratory system and blue cells indicate cell signatures of respiratory system associated immune cells. GSEA; Gene Set Enrichment Analysis and EAC: Oesophageal Adenocarcinoma.

**Additional file 13: Table S5.** D) Table indicating correlation coefficients and their associated *p*-value for correlation between two genes for Stage IV interactions. The cells highlighted with bold blue text indicate associations that have been proved experimentally. Additionally, the grey color cells indicate significant associations only in either pearson or spearman correlation coefficient, while the rest of the values are significant for both correlation coefficients.

**Additional file 14: Table S6.** B) Network Parameters and their respective ranks computed for Stage I GRNs ON, DD and DP. Pseudogenes are highlighted in dark yellow while transcription factors are highlighted in light yellow. The genes with an above-average rank are higlighted in bold. GRN; Gene Regulatory Network, ON; Original Network, DD; Degree of Differentiation and DP; Degree of Pleiotropy.

**Additional file 15: Table S7.** Table indicating the hazard's ratio and their respective *p*-values obtained from coxph regression for oesophageal carcinoma (ESCA) and stage II alone (Stage II ESCA). N represents the number of samples with above median (high) or below median (low) gene expression as the variable. For hazard's ratio (HR), <1 indicates low risk and >1 indicates high risk to survival with respect to the reference for a given variable. The 95% confidence intervals for HR are represented in the bracket along with respective *p*-values, where *p*-values ≤ 0.05 are highlighted in bold italic.

## Data Availability

All datasets analysed are publicly available as indicated in the manuscript.
