# Supplementary material for: Stage II oesophageal carcinoma: peril in disguise associated with cellular reprogramming and oncogenesis regulated by pseudogenes
Source: BMC Genomics. 2024 Feb 2;25:135. doi: 10.1186/s12864-024-10023-9 (PMC10835973; doi:10.1186/s12864-024-10023-9)
Supplement: Supplementary file 1 — Additional file 1: Figure S1. Subset of DaPs exhibit Stage-specific expression pattern across ESCA Box and whisker plots indicating the gene expression profile of DaPs differentially expressed in Stage I (a), Stage II (c), Stage III (e) and Stage IV (g) ESCA as compared to that of normal. Significance of expression between the two groups for each DaP was evaluated using Mann-Whitney tests; indicated by asterisks above the boxplot as *p < 0.05, **p < 0.01 and ns (non-significant). Volcano plots of differentially expressed genes in Stage I (b), Stage II (d), Stage III (f) and Stage IV (h) ESCA indicating down-regulated and up-regulated DaPs shown as green and red dots, respectively. The cut-off for |log2 FC| ≥ 1.5 is indicated by dashed-lines along x-axis, while the dashed-line along y-axis indicates cut-off for -log10(adjusted p-value) > 1.30103. Additionally, stage-specific downregulation of DaPs is indicated by orange dots, while pale-green dots represent stage-specific upregulation. Genes with adjusted p-value <-log10(0.05) and/or |log2FC| < 1.5 are indicated by grey dots. DaPs; Differentiation-associated pseudogenes, FC; Fold Change and ESCA; Oesophageal Carcinoma. [file 12864_2024_10023_MOESM1_ESM.docx]

**Figure S1. Subset of DaPs exhibit Stage-specific expression pattern across ESCA** Box and whisker plots indicating the gene expression profile of DaPs differentially expressed in Stage I (a), Stage II (c), Stage III (e) and Stage IV (g) ESCA as compared to that of normal. Significance of expression between the two groups for each DaP was evaluated using Mann-Whitney tests; indicated by asterisks above the boxplot as *p < 0.05, **p < 0.01 and ns (non-significant). Volcano plots of differentially expressed genes in Stage I (b), Stage II (d), Stage III (f) and Stage IV (h) ESCA indicating down-regulated and up-regulated DaPs shown as green and red dots, respectively. The cut-off for |log2 FC| ≥ 1.5 is indicated by dashed-lines along x-axis, while the dashed-line along y-axis indicates cut-off for -log10(adjusted p-value) > 1.30103. Additionally, stage-specific downregulation of DaPs is indicated by orange dots, while pale-green dots represent stage-specific upregulation. Genes with adjusted p-value < -log10(0.05) and/or |log2FC| < 1.5 are indicated by grey dots. DaPs; Differentiation-associated pseudogenes, FC; Fold Change and ESCA; Oesophageal Carcinoma
