# Supplementary material for: Stage II oesophageal carcinoma: peril in disguise associated with cellular reprogramming and oncogenesis regulated by pseudogenes
Source: BMC Genomics. 2024 Feb 2;25:135. doi: 10.1186/s12864-024-10023-9 (PMC10835973; doi:10.1186/s12864-024-10023-9)
Supplement: Supplementary file 2 — Additional file 2: Figure S2. PiGs, DaGs and miRNAs exhibit unique expression pattern similar to that of DaPs across ESCA a-l) Volcano plots associated with Stage I (a-c), Stage II (d-f), Stage III (g-i) and Stage IV (j-l) ESCA indicating differentially expressed PiGs (a, d, g and j for Stage I, II, III and IV, respectively), DaGs (b, e, h and k for Stage I, II, III and IV, respectively) and miRNAs (c, f, i and l for Stage I, II, III and IV, respectively). PiGs and their interactions with DaPs across each stage are labelled as ‘PiG-DaP’ combination for their respective dots using a line. Green dots indicate down-regulation, while red dots indicate up-regulation for PiGs and DaCGs. Similarly, for miRNAs, downregulation is indicated by green diamonds, while upregulation is indicated by red diamonds. Dashed-lines along x-axis indicate the cut-off for|log2 FC| ≥ 1.5, while the dashed-line along y-axis indicates cut-off for-log10(adjusted p-value) > 1.30103. Additionally, for PiGs and DaGs, stage-specific downregulation is indicated by orange dots, while pale-green dots represent stage-specific upregulation. Similarly, for miRNAs, orange diamonds indicate stage-specific downregulation, while pale-green diamonds indicate stage-specific upregulation. Yellow dots and diamonds indicate de-regulation across all stages of ESCA, referred to as ‘Constitutively De-regulated’ for PiGs and miRNAs, respectively. Lavender diamonds indicate miRNAs de-regulated across more than one stage, with the stages of ESCA indicated in the brackets in their respective box of legends. Genes with adjusted p-value <-log10(0.05) and/or |log2FC| < 1.5 are indicated by grey dots for PiGs and DaGs, while for miRNAs they are represented using grey diamonds. DaPs; Differentiation-associated pseudogenes and ESCA; Oesophageal Carcinoma. [file 12864_2024_10023_MOESM2_ESM.docx]

**Figure S2. PiGs, DaGs and miRNAs exhibit unique expression pattern similar to that of DaPs across ESCA** a-l) Volcano plots associated with Stage I (a-c), Stage II (d-f), Stage III (g-i) and Stage IV (j-l) ESCA indicating differentially expressed PiGs (a, d, g and j for Stage I, II, III and IV, respectively), DaGs (b, e, h and k for Stage I, II, III and IV, respectively) and miRNAs (c, f, i and l for Stage I, II, III and IV, respectively). PiGs and their interactions with DaPs across each stage are labelled as ‘PiG-DaP’ combination for their respective dots using a line. Green dots indicate down-regulation, while red dots indicate up-regulation for PiGs and DaCGs. Similarly, for miRNAs, downregulation is indicated by green diamonds, while upregulation is indicated by red diamonds. Dashed-lines along x-axis indicate the cut-off for |log2 FC| ≥ 1.5, while the dashed-line along y-axis indicates cut-off for -log10(adjusted p-value) > 1.30103. Additionally, for PiGs and DaGs, stage-specific downregulation is indicated by orange dots, while pale-green dots represent stage-specific upregulation. Similarly, for miRNAs, orange diamonds indicate stage-specific downregulation, while pale-green diamonds indicate stage-specific upregulation. Yellow dots and diamonds indicate de-regulation across all stages of ESCA, referred to as ‘Constitutively De-regulated’ for PiGs and miRNAs, respectively. Lavender diamonds indicate miRNAs de-regulated across more than one stage, with the stages of ESCA indicated in the brackets in their respective box of legends. Genes with adjusted p-value < -log10(0.05) and/or |log2FC| < 1.5 are indicated by grey dots for PiGs and DaGs, while for miRNAs they are represented using grey diamonds. DaPs; Differentiation-associated pseudogenes and ESCA; Oesophageal Carcinoma
