# Supplementary material for: Stage II oesophageal carcinoma: peril in disguise associated with cellular reprogramming and oncogenesis regulated by pseudogenes
Source: BMC Genomics. 2024 Feb 2;25:135. doi: 10.1186/s12864-024-10023-9 (PMC10835973; doi:10.1186/s12864-024-10023-9)
Supplement: Supplementary file 3 — Additional file 3: Figure S3. Distinct Biological Processes, KEGG and Immune Signatures are enriched for de-regulated PiGs and DaCGsa) LEA plots (left) indicating the correlation between the enriched biological processes for Stage I ESCA with the corresponding expression heatmap of LEA genes (right). b-c) LEA plots (left) indicating the correlation between the enriched biological processes (b) and KEGG pathways (c) for Stage II ESCA and their respective heatmaps indicating expression of LEA genes (right). d) Heatmap indicating expression of LEA genes enriched for biological processes across Stage III ESCA e) LEA plot (left) indicating the correlation between the enriched immune signatures for Stage III ESCA and respective heatmap indicating expression of LEA genes (right). (f) LEA plot (top) indicating the correlation between enriched biological processes for Stage IV ESCA with the corresponding expression of LEA genes (bottom). For all heatmaps, blue indicates downregulation while red indicates upregulation in cancer samples with respect to normal. KEGG; Kyoto Encyclopaedia of Genes and Genomes, PiGs; DaP-interacting genes, DaP; Differentiation-associated Pseudogenes, DaCGs; Differentiation-associated Coding Genes, LEA; Leading Edge Analysis and ESCA; Oesophageal Carcinoma. [file 12864_2024_10023_MOESM3_ESM.docx]

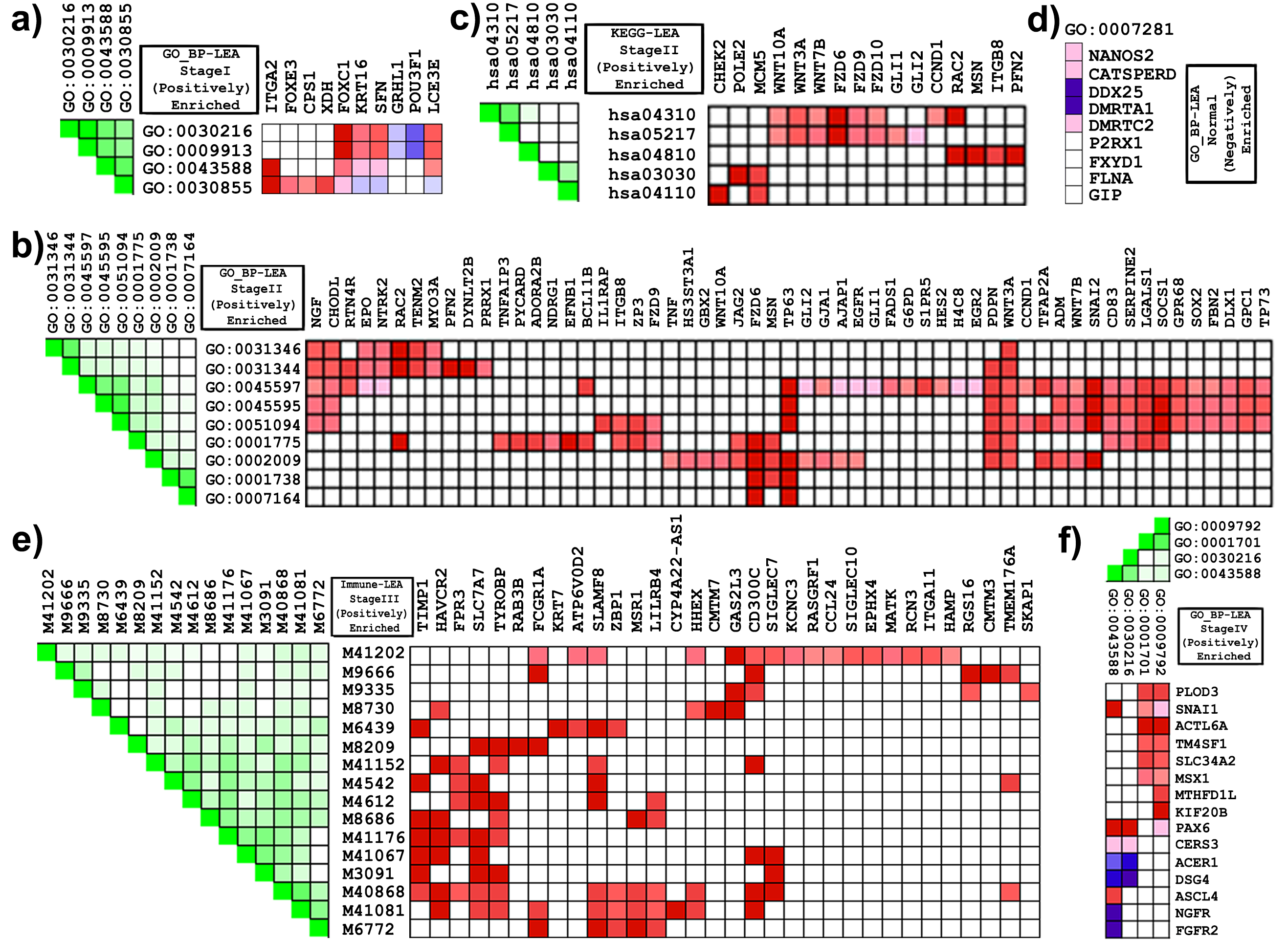


**Figure S3: Distinct Biological Processes, KEGG and Immune Signatures are enriched for de-regulated PiGs and DaCGs** a) LEA plots (left) indicating the correlation between the enriched biological processes for Stage I ESCA with the corresponding expression heatmap of LEA genes (right). b-c) LEA plots (left) indicating the correlation between the enriched biological processes (b) and KEGG pathways (c) for Stage II ESCA and their respective heatmaps indicating expression of LEA genes (right). d) Heatmap indicating expression of LEA genes enriched for biological processes across Stage III ESCA e) LEA plot (left) indicating the correlation between the enriched immune signatures for Stage III ESCA and respective heatmap indicating expression of LEA genes (right). (f) LEA plot (top) indicating the correlation between enriched biological processes for Stage IV ESCA with the corresponding expression of LEA genes (bottom). For all heatmaps, blue indicates downregulation while red indicates upregulation in cancer samples with respect to normal. KEGG; Kyoto Encyclopaedia of Genes and Genomes, PiGs; DaP-interacting genes, DaP; Differentiation-associated Pseudogenes, DaCGs; Differentiation-associated Coding Genes, LEA; Leading Edge Analysis and ESCA; Oesophageal Carcinoma
