# Supplementary material for: Stage II oesophageal carcinoma: peril in disguise associated with cellular reprogramming and oncogenesis regulated by pseudogenes
Source: BMC Genomics. 2024 Feb 2;25:135. doi: 10.1186/s12864-024-10023-9 (PMC10835973; doi:10.1186/s12864-024-10023-9)
Supplement: Supplementary file 4 — Additional file 4: Figure S4. Expression-Independent Enrichment landscape of differentially expressed PiGs and LEA-DaCGs across various stages of ESCA a-f) Pie charts indicating the distribution of enriched terms obtained using ShinyGO across various stages of ESCA for biological processes (a), cellular components (b), molecular functions (c), KEGG pathways (d), hallmarks (e) and wiki-pathways (f). a1-f4) Chord diagrams indicating the association between the stage-specific and “constitutively” enriched biological processes (a1-a4), cellular components (b1-b4), molecular functions (c1-c4), KEGG pathways (d1-d3), hallmarks (e1-e3) and wiki-pathways (f1-f4). Enrichments exhibiting “constitutive” pattern are highlighted in purple while stage-specific enrichment is highlighted in black. Nodes are indicated in blue with more significantly enriched terms as dark blue and a decrease in significance indicated as a decrease in the colour. The association between nodes is represented using red edges where bright red indicates higher overlap of genes and a decrease in overlap is indicated by decrease in the colour. [file 12864_2024_10023_MOESM4_ESM.docx]

**Figure S4: Expression-Independent Enrichment landscape of differentially expressed PiGs and LEA-DaCGs across various stages of ESCA** a-f) Pie charts indicating the distribution of enriched terms obtained using ShinyGO across various stages of ESCA for biological processes (a), cellular components (b), molecular functions (c), KEGG pathways (d), hallmarks (e) and wiki-pathways (f). a1-f4) Chord diagrams indicating the association between the stage-specific and “constitutively” enriched biological processes (a1-a4), cellular components (b1-b4), molecular functions (c1-c4), KEGG pathways (d1-d3), hallmarks (e1-e3) and wiki-pathways (f1-f4). Enrichments exhibiting “constitutive” pattern are highlighted in purple while stage-specific enrichment is highlighted in black. Nodes are indicated in blue with more significantly enriched terms as dark blue and a decrease in significance indicated as a decrease in the colour. The association between nodes is represented using red edges where bright red indicates higher overlap of genes and a decrease in overlap is indicated by decrease in the colour.
