# Supplementary material for: Stage II oesophageal carcinoma: peril in disguise associated with cellular reprogramming and oncogenesis regulated by pseudogenes
Source: BMC Genomics. 2024 Feb 2;25:135. doi: 10.1186/s12864-024-10023-9 (PMC10835973; doi:10.1186/s12864-024-10023-9)
Supplement: Supplementary file 5 — Additional file 5: FigureS5. GRNs indicating the association between genes for all stages of ESCA Gene regulatory networks indicating the interactions between DaPs, PiGs, DaCGs and miRNAs for Degree of Differentiation or DD (a) and Degree of Pleiotropy or DP (b). DD represents interactions between DaPs, miRNAs and genes identified as part of LEA, while DP represents interactions between DaPs, miRNAs and genes having functional pleiotropy. The node types and their respective colours within a given stage are indicated in the bottom-right legend. Black edges are evidence-based interactions curated from studies that have validated these interactions experimentally, while grey edges indicate potential interactions. All interactions have a significant p-value for either Spearman or Pearson correlation coefficient, or both. Thin edges represent a significant p-value for only one correlation coefficient. Denser edges represent a significantp-value for both correlation coefficients, where the density is moderate for absolute value of coefficients < 0.89 and highest for absolute value of ≥ 0.89 coefficient. GRNs; Gene regulatory network, DaPs; Differentiation-associated pseudogenes, PiGs; DaP-interacting genes, DaCGs; Differentiation-associated coding genes, TFs; Transcription factors and ESCA; Oesophageal Carcinoma. [file 12864_2024_10023_MOESM5_ESM.docx]

**

**

**FigureS5: GRNs indicating the association between genes for all stages of ESCA** Gene regulatory networks indicating the interactions between DaPs, PiGs, DaCGs and miRNAs for Degree of Differentiation or DD (a) and Degree of Pleiotropy or DP (b). DD represents interactions between DaPs, miRNAs and genes identified as part of LEA, while DP represents interactions between DaPs, miRNAs and genes having functional pleiotropy. The node types and their respective colours within a given stage are indicated in the bottom-right legend. Black edges are evidence-based interactions curated from studies that have validated these interactions experimentally, while grey edges indicate potential interactions. All interactions have a significant p-value for either Spearman or Pearson correlation coefficient, or both. Thin edges represent a significant p-value for only one correlation coefficient. Denser edges represent a significant p-value for both correlation coefficients, where the density is moderate for absolute value of coefficients < 0.89 and highest for absolute value of ≥ 0.89 coefficient. GRNs; Gene regulatory network, DaPs; Differentiation-associated pseudogenes, PiGs; DaP-interacting genes, DaCGs; Differentiation-associated coding genes, TFs; Transcription factors and ESCA; Oesophageal Carcinoma
