# Supplementary material for: Stage II oesophageal carcinoma: peril in disguise associated with cellular reprogramming and oncogenesis regulated by pseudogenes
Source: BMC Genomics. 2024 Feb 2;25:135. doi: 10.1186/s12864-024-10023-9 (PMC10835973; doi:10.1186/s12864-024-10023-9)
Supplement: Supplementary file 6 — Additional file 6: Figure S6. SOX2 regulates a subset of stage II-specific DaPs and DaCGs MAST results indicating motif alignment of de novo STREME9 motif identified from relative enrichment of motifs in DaPs as compared to that of DaCGs and PiGs having putative or known interactions with stage II TFs. STREME9 motif alignment across stage II-specific DaPs (a-f, i, l-o) and DaCGs (p) as well as constitutively de-regulated DaPs (g-h, j-k) along with their respective p-values, sequence alignment range and strand direction. The horizontal bar beside the gene name indicates gene sequence length (462bp for DaPs and 4000bp; truncated to 462bp for DaCGs) with the left vertical bar indicating the start of visual range of motif alignment while the right vertical bar indicates the end of visual range. The plus and minus sign alongside the sequence indicates strand direction with the red vertical bars indicating the aligned motifs. Motif on positive strand indicates normal motif, while motif on negative strand indicates reverse complement. The individual p-valuesassociated with motif is indicated above the aligned motif, while the overall p-values are indicated along with the gene names. [file 12864_2024_10023_MOESM6_ESM.docx]

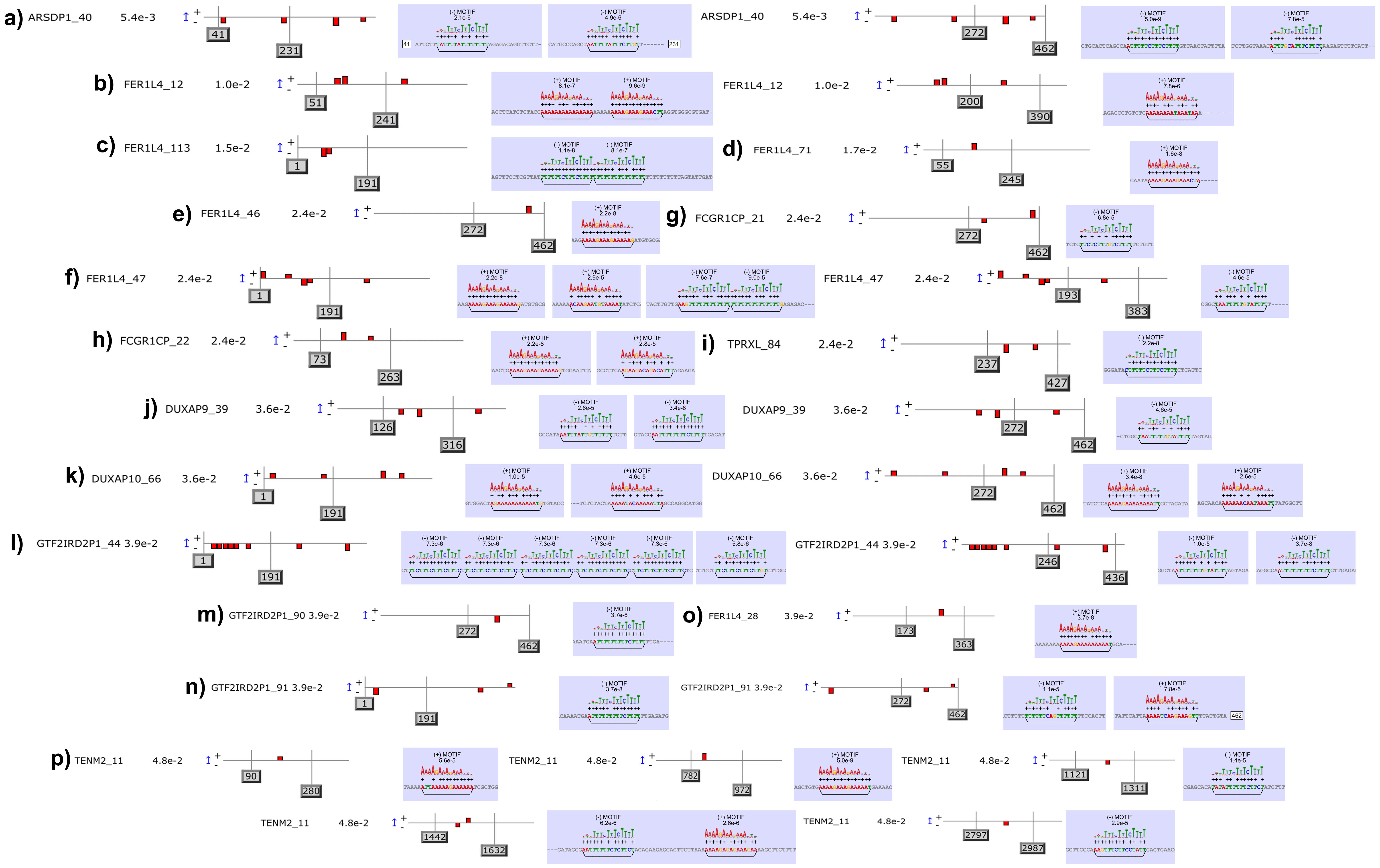


**Figure S6: *SOX2* regulates a subset of stage II-specific DaPs and DaCGs** MAST results indicating motif alignment of *de novo* STREME9 motif identified from relative enrichment of motifs in DaPs as compared to that of DaCGs and PiGs having putative or known interactions with stage II TFs. STREME9 motif alignment across stage II-specific DaPs (a-f, i, l-o) and DaCGs (p) as well as constitutively de-regulated DaPs (g-h, j-k) along with their respective *p-values*, sequence alignment range and strand direction. The horizontal bar beside the gene name indicates gene sequence length (462bp for DaPs and 4000bp; truncated to 462bp for DaCGs) with the left vertical bar indicating the start of visual range of motif alignment while the right vertical bar indicates the end of visual range. The plus and minus sign alongside the sequence indicates strand direction with the red vertical bars indicating the aligned motifs. Motif on positive strand indicates normal motif, while motif on negative strand indicates reverse complement. The individual *p-values* associated with motif is indicated above the aligned motif, while the overall *p-values* are indicated along with the gene names.
