# Supplementary material for: Stage II oesophageal carcinoma: peril in disguise associated with cellular reprogramming and oncogenesis regulated by pseudogenes
Source: BMC Genomics. 2024 Feb 2;25:135. doi: 10.1186/s12864-024-10023-9 (PMC10835973; doi:10.1186/s12864-024-10023-9)
Supplement: Supplementary file 7 — Additional file 7: Figure S7. Unique Mutational Landscape of Combinatorial de-regulation of DaPsa) Rainfall plot indicating the events of kataegis (green star) across the chromosomes 1-22 and X (x-axis) for entire ESCA (a), each stage of ESCA (b) and samples stratified based on the median cut-off of APOBEC gene expression (c). Kataegis is represented by a green star along (a-c) with the average inter-mutation distance as obtained from MAF tools (c). The class of single-base substitutions that constitute the hypermutations are represented below the plot. The APOBEC genes that are upregulated are represented in lavender (a-b) with the extent of differential expression as Log2FC and respective p-adjusted values (b). The class of single-base substitutions that constitute the hypermutations are represented below the plot d) An elbow plot indicating the number of signatures as clusters on x-axis generated through unsupervised learning, non-negative matrix factorization. The number of signatures is decided by a significant drop in the correlation coefficient.ll plot indicating the events of kataegis (green star) across the chromosomes 1-22 and X (x-axis) for entire ESCA (a), each stage of ESCA (b) and samples stratified based on the median cut-off of APOBEC gene expression (c). Kataegis is represented by a green star along (a-c) with the average inter-mutation distance as obtained from MAF tools (c). The class of single-base substitutions that constitute the hypermutations are represented below the plot. The APOBEC genes that are upregulated are represented in lavender (a-b) with the extent of differential expression as Log2FC and respective p-adjusted values (b). The class of single-base substitutions that constitute the hypermutations are represented below the plot d) An elbow plot indicating the number of signatures as clusters on x-axis generated through unsupervised learning, non-negative matrix factorization. The number of signatures is decided by a significant drop [file 12864_2024_10023_MOESM7_ESM.docx]

**Figure S7: Unique Mutational Landscape of Combinatorial de-regulation of DaPs** a) Rainfall plot indicating the events of kataegis (green star) across the chromosomes 1-22 and X (x-axis) for entire ESCA (a), each stage of ESCA (b) and samples stratified based on the median cut-off of APOBEC gene expression (c). Kataegis is represented by a green star along (a-c) with the average inter-mutation distance as obtained from MAF tools (c). The class of single-base substitutions that constitute the hypermutations are represented below the plot. The APOBEC genes that are upregulated are represented in lavender (a-b) with the extent of differential expression as Log2FC and respective *p-adjusted values* (b). The class of single-base substitutions that constitute the hypermutations are represented below the plot d) An elbow plot indicating the number of signatures as clusters on x-axis generated through unsupervised learning, non-negative matrix factorization. The number of signatures is decided by a significant drop in the correlation coefficient.
