# Supplementary material for: Stage II oesophageal carcinoma: peril in disguise associated with cellular reprogramming and oncogenesis regulated by pseudogenes
Source: BMC Genomics. 2024 Feb 2;25:135. doi: 10.1186/s12864-024-10023-9 (PMC10835973; doi:10.1186/s12864-024-10023-9)
Supplement: Supplementary file 8 — Additional file 8: Figure S8. Stage II ESCA may act as potential Tipping Point for Differentiation induced Oncogenesis Kaplan-Meier plots indicating survival probability over increasing time for a) all stages of ESCA b) varied combinatorial expression of FEV and KLF15for SII UDaP_No_Combo and c) patient stratified Stage II and tumours ≥ Stage III. The tables below each graph indicate risk table (top) and censored population over time (bottom). The number of patients at risk is indicated in numbers with the respective percentages in the bracket for risk table. The censored observations for each variable are indicated by their respective colour for censored population table. ESCA; Oesophageal Carcinoma, SII; Stage II, UDaP_Combo; Upregulated& downregulated DaPs Combination and UDaP_No_Combo; Upregulated& downregulated DaPs No Combination. [file 12864_2024_10023_MOESM8_ESM.docx]

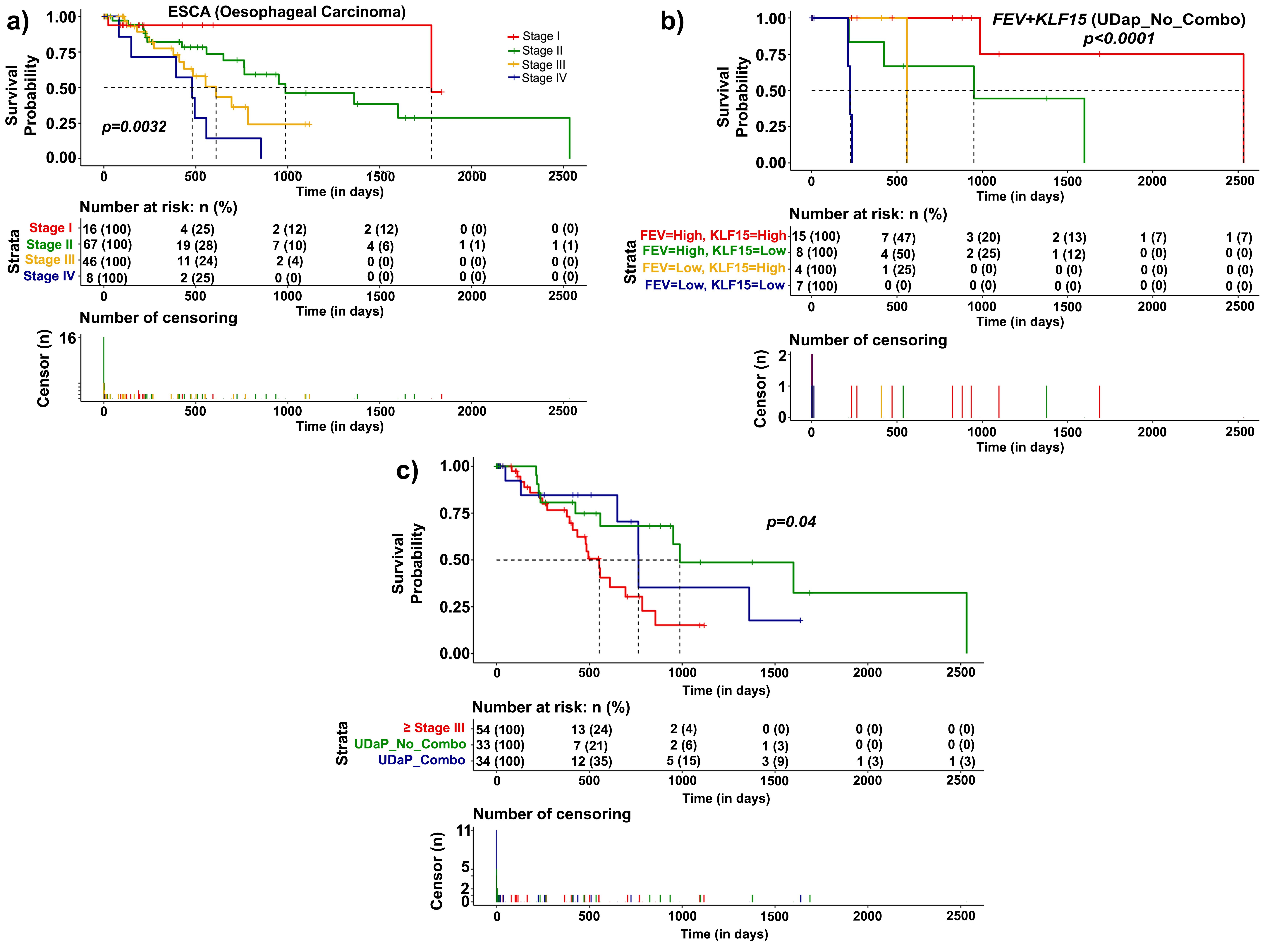


**Figure S8: Stage II ESCA may act as potential Tipping Point for Differentiation induced Oncogenesis** Kaplan-Meier plots indicating survival probability over increasing time for a) all stages of ESCA b) varied combinatorial expression of *FEV* and *KLF15* for SII UDaP_No_Combo and c) patient stratified Stage II and tumours ≥ Stage III. The tables below each graph indicate risk table (top) and censored population over time (bottom). The number of patients at risk is indicated in numbers with the respective percentages in the bracket for risk table. The censored observations for each variable are indicated by their respective colour for censored population table. ESCA; Oesophageal Carcinoma, SII; Stage II, UDaP_Combo; **U**pregulated & downregulated **DaP**s **Comb**inati**o**n and UDaP_No_Combo; **U**pregulated & downregulated **DaP**s No **Comb**inati**o**n
